# *Arthroaspis* n. gen., a common element of the Sirius Passet Lagerstätte (Cambrian, North Greenland), sheds light on trilobite ancestry

**DOI:** 10.1186/1471-2148-13-99

**Published:** 2013-05-11

**Authors:** Martin Stein, Graham E Budd, John S Peel, David AT Harper

**Affiliations:** 1 Natural History Museum of Denmark, Universitetsparken 15, DK-2100 Copenhagen, Denmark; 2 Department of Earth Sciences, Palaeobiology, Uppsala University, Villavägen 16, SE 752 36 Uppsala, Sweden; 3 Department of Earth Sciences, Durham University, Science Labs, Durham DH1 3LE, UK

**Keywords:** Sirius Passet Lagerstätte, Arthropoda, Lamellipedia, Trilobita, Appendages, Pygidium, Functional morphology

## Abstract

**Background:**

Exceptionally preserved Palaeozoic faunas have yielded a plethora of trilobite-like arthropods, often referred to as lamellipedians. Among these, Artiopoda is supposed to contain taxa united by a distinctive appendage structure. This includes several well supported groups, Helmetiida, Nektaspida, and Trilobita, as well as a number of problematic taxa. Interrelationships remain unclear, and the position of the lamellipedian arthropods as a whole also remains the subject of debate.

**Results:**

*Arthroaspis bergstroemi* n. gen. n. sp., a new arthropod from the early Cambrian Sirius Passet Lagerstätte of North Greenland shows a striking combination of both dorsal and ventral characters of Helmetiida, Nektaspida, and Trilobita. Cladistic analysis with a broad taxon sampling of predominantly early Palaeozoic arthropods yields a monophyletic Lamellipedia as sister taxon to the Crustacea or Tetraconata. Artiopoda is resolved as paraphyletic, giving rise to the Marrellomorpha. Within Lamellipedia, a clade of pygidium bearing taxa is resolved that can be shown to have a broadly helmetiid-like tergite morphology in its ground pattern. This morphology is plesiomorphically retained in Helmetiida and in *Arthroaspis*, which falls basally into a clade containing Trilobita. The trilobite appendages, though similar to those of other lamellipedians in gross morphology, have a unique outward rotation of the anterior trunk appendages, resulting in a ‘hard wired’ lateral splay, different to that observed in other Lamellipedia.

**Conclusions:**

The combination of helmetiid, trilobite, and nektaspid characters in *Arthroaspis* gives important hints concerning character polarisation within the trilobite-like arthropods. The distinctive tergite morphology of trilobites, with its sophisticated articulating devices, is derived from flanged edge-to-edge articulating tergites forming a shield similar to the helmetiids, previously considered autapomorphic for that group. The stereotypical lateral splay of the appendages of lamellipedians is a homoplastic character shown to be achieved by several groups independently.

## Background

Arthropod diversity in the Cambrian is now reasonably well-known through the discovery and description of the faunas from the Chengjiang, Emu Bay Shale, and Sirius Passet Lagerstätten in the early Cambrian, the Burgess Shale and similar deposits in the middle Cambrian, and the Orsten-type deposits, mostly from the late Cambrian. When the true diversity of these taxa became clear in the 1970s, their unfamiliarity prompted the view that they represented the end points of many independent lines of evolution that had convergently undergone arthropodisation [1]. However, this view was relatively short-lived in the advent of rigorous cladistic analysis [2,3], and the essential similarity between many of these taxa began to be recognised [4]. The first full-blown cladistic analysis of the Burgess Shale taxa was that of D. E. G. Briggs and R. A. Fortey [5], which suggested that most Cambrian arthropods lay within the stem group of Chelicerata, and that the trilobites were relatively derived. An important exception to this view were the arthropods of the Orsten fauna, some of which have been shown convincingly to lie in the stem- and crown-group of Crustacea or Tetraconata [6-10].

More recently, the view that some Cambrian arthropods lie outside the crown-group of Euarthropoda has gained ground [11-13]. These taxa include, for example, *Fuxianhuia protensa* Hou, 1987, *Chengjiangocaris longiformis* Hou & Bergström, 1991, and *Shankouia zhenghei* Chen, Wang, Maas & Waloszek in Waloszek *et al*., 2005. Whether taxa such as *Canadaspis perfecta* (Walcott, 1912) are derivatives of the euarthropod stem-lineage [14-16] or represent the crown-group [17] is still a matter of debate. Another open debate is whether Megacheira, including taxa such as *Leanchoilia superlata* Walcott, 1912 and *Yohoia tenuis* Walcott, 1912 fall outside Euarthropoda [12,14,16,18,19] or are derivatives of the early chelicerate stem-lineage [20-24].

There is consensus that Trilobita belongs to Euarthropoda, but there are two competing hypotheses for their phylogenetic position within the monophylum. The traditional Arachnomorpha [20,22,25-27] is being increasingly questioned, and an increasing number of studies suggest trilobites to be derivatives of the mandibulatan stem-lineage [16,19,28-31]. Regardless, there is consensus that trilobites belong to a larger group of early Paleozoic arthropods, containing many of the non-biomineralizing taxa attributed to Arachnomorpha. They share the presence of distinctive lamellar blades on their exopods, and X-g. Hou and J. Bergström proposed Lamellipedia to embrace them [12]. In its original concept, Lamellipedia was a grade comprising the stem-lineage of Chelicerata, including the non-chelicerate arachnomorphs except for Megacheira [12]. Within Lamellipedia, the basal split set off Marrellomorpha from Artiopoda, which comprised the remainder of the chelicerate stem-lineage. Some authors have suggested that Artiopoda could be a monophylum based on the shared presence of a rather similar limb with a bilobate exopod of a proximal lobe bearing the lamellae and a flap-like distal lobe fringed with setae [28] and indeed they were rendered monophyletic in some analyses [16,19,31]. While most analyses yield a number of well supported groups of artiopodans, in particular Helmetiida, Nekatspidida, Trilobita, and Xandarellida, their interrelationships remain unclear [19,22,26,27,31,32].

In summary, then, whereas several groups of Cambrian arthropods are clearly related, their overall relationships remain uncertain. Furthermore, the monophyletic or paraphyletic status of several of these groupings remains in question. Whilst this debate will not be resolved quickly – it depends partly on differences in philosophy of systematics – the further description of exceptionally-preserved taxa will aid its resolution. Here, then, we describe a new exceptionally-preserved lamellipedian arthropod from the Sirius Passet Lagerstätte that adds significant new data to the debate, and that also has a bearing on the ecology of these early arthropods.

### The Sirius Passet Lagerstätte

The discovery of the Sirius Passet Lagerstätte was announced by S. Conway Morris, J. S. Peel, A. K. Higgins, N. J. Soper, and N. C. Davis [33] and has been recounted by S. Conway Morris [34] and J. S. Peel and J. R. Ineson [35]. The fossiliferous strata crop out in north-west Peary Land along the southern slopes of the broad valley which gives its name to the locality and just south of the mountainous northern rim (Nansen Land) of Greenland (lat. 82°47.6´N, long. 42°13.7´W); it is the most remote and least well known of the classic Cambrian Explosion biotas. The lagerstätte is of early Cambrian age, (Cambrian Series 2, Montezuman Stage of Laurentian usage [36]).

The geological setting was described by J. S. Peel and J. R. Ineson [35,37]; it is now known that the deposits yielding exceptional preservation are located within a 14 m interval of the Buen Formation at the main locality [38] and fossils are especially common in the middle part of the section. However, the relationship of other fossiliferous localities in the immediate vicinity to the main locality is uncertain [38]. Since its discovery in 1984, expeditions in 1985, 1989, 1991, 1994 and 2006, partly under the auspices of the Geological Survey of Greenland, have assembled almost 10 000 specimens mainly from talus below the main outcrop [38]. More than 30 species have been described in detail to date [38,39] and the fauna is dominated by arthropods, lobopods and cycloneuralian and polychaete worms. New collections made during expeditions from the Natural History Museum, University of Copenhagen, in 2009 and 2011 focussed on *in situ* material from the main exposure of the Transitional Buen Formation at the Sirius Passet locality [40,41] and have allowed the mapping of faunal associations within the formation.

The Buen Formation crops out across a large part of North Greenland [42,43]. Its siliciclastic sediments represent the expansion of a northern basin across a subsiding carbonate platform to the south within the trans-arctic Franklinian Basin succession. In southern areas the formation is dominated by sandstones but the proportion of coarse-grained siliciclastics decreases northwards into the deeper-water parts of the basin. In the vicinity of the Sirius Passet locality, a narrow belt (200 – 1400 m wide) of mainly fine-grained siliciclastic sediments forms a transitional zone between the southern shelf succession and the equivalent deep-water basinal deposits of the Polkorridoren Group to the north [35,42]. This so-called Transitional Buen Formation includes the fossiliferous deposits of the Sirius Passet Lagerstätte [35].

Bioturbation, indicated by mottling, is pervasive throughout much of the Transitional Buen Formation except for the lagerstätte intervals, where the lamination is strongly developed, showing partings at a 3–10 mm scale. A depositional setting on the outer parts of the shelf to upper slope, possibly dominated by suspension fallout material [35,42] is envisaged.

The understanding of the preservational processes in the Sirius Passet Lagerstätte is far less advanced than those for the Burgess [44-46] and Chengjiang [47] lagerstätten. The preservation of the Greenland fossils appears to differ significantly from that of other Cambrian exceptionally-preserved faunas, with early mineralisation appearing to play an important role [48,49]. However, preservation within the Sirius Passet Lagerstätte is variable. Mineralised forms such as trilobites, hyolithids and articulated halkieriids preserve much of their relief while even un-mineralised organisms retain a measure of 3-dimensional form. At certain horizons relief is lost and organisms are preserved as reflective films. The Sirius Passet fossils are often visually unimpressive when compared to those from Chengjiang or the Burgess Shale. It is characteristic that central regions of many fossils, mainly the body cavity, tend to be best preserved in contrast to the poor definition of body outlines (e.g. *Kerygmachela kierkegaardi* Budd, 1993 [50], *Pambdelurion whittingtoni* Budd, 1997 [51], and *Campanamuta mantonae* Budd, 2011 [52]; preferential preservation of the central regions is possibly associated with high concentrations of carbon [53]. As a result, taphonomic insights gained from other faunas should only be applied with caution to these fossils. This can make comparison of these to more conventionally-preserved taxa difficult. In addition the high metamorphic grade, illustrated by abundant acicular chlorite crystals within the sediments, adds a further dimension to the understanding of a complex set of taphonomic pathways.

The new taxon is large (some specimens exceed 20 cm in length). Specimens, as preserved, appear generally robust with good relief and show few wrinkles, and are largely complete although isolated thoracic tergites, limbs and cephalic and pygidial shields are not uncommon. The majority of the specimens appear to have been preserved *in situ*, with little or no evidence of transport and the wide range of instars confirms the remains of a census population.

The specimens of the new taxon are generally characterised by a network of complex, interconnected burrow systems constructed by a meiofauna and small macrofauna of invertebrates [36,37,39,54]. Moreover apart from providing an oasis of primary nutrients in an otherwise poorly-resourced landscape, high sulphate concentrations may have encouraged the colonization of the burrows by sulphur bacteria, providing a secondary food source [54]. The slowly decaying carcasses clearly attracted invertebrate communities that both mined and farmed the burrow systems.

Initial impressions of the sediments suggest deposition within dysoxic environments; nevertheless specimens of the new taxon are preserved with a post-mortem, commensal epifauna of small invertebrates. The dark shales within the lagertätte intervals are, however, punctuated by thin partings of coarser sediment, which suggest a periodic freshening of the sediment-water interface and the potential to transport living meiofauna into deeper-water environments where they could profitably engage with the *Arthroaspis* carcasses. The proposed presence of sulphate and sulphur bacteria [54] suggests oxygen deprivation that would inhibit destructive, microbial activity [47] and the coarser-grained sediments, the distal products from shelfal storms, subsequently entombed the large arthropods in obrution deposits. The large *Arthroaspis* clearly thrived in conditions at the sediment-water interface but its preservation was aided by local dysoxic conditions within the sediment and the cover of coarser sediments derived from upslope.

## Methods

Where required, specimens were prepared with a pneumatic chisel. All specimens were cleaned with an ultrasonic bath and coated with colloidal graphite that was applied with an air brush. Prior to photography specimens were coated with ammonium chloride. Photographs were taken under low-angle light.

The description in general refers to large specimens that represent holaspids; morphometric change throughout holaspid ontogeny was not studied in detail. The post-antennular limbs are not sufficiently well-known to allow their individual description, although a general understanding of their structure is possible.

Repositories: MGUH, Natural History Museum of Denmark, Copenhagen, Denmark; USNM, National Museum of Natural History, Smithsonian Institution, Washington, DC.

### Terminology

Terminology follows standard trilobite terminology [55] complemented with descriptive terminology applied to exceptionally preserved arthropods from the Orsten and Burgess Shale Lagerstätten [31,56]. Deviating from these works, the term trunk in the following refers to the thorax and pygidium combined, and hypostomal complex to the hypostome and prehypostomal sclerite. Abbreviations used in the figures are summarized in Table 1.

**Table 1:** Abbreviations used in figures

*af*	axial furrow
*afl*	articulating flange
*ahr*	articulating half ring
*an*	axial node
*ar1–3*	pygidial axial rings
*atl*	antennula
*aw*	anterior wings of hypostome
*ba*	basipod
*blj*	body–limb joint
*Cgd*	midgut diverticula of the cephalon
*C1–3s*	sternites of cephalic segments
*C/T*	cephalothoracic boundary
*d*	doublure
*dar*	doublure of axial ring
*dm*	doublural margin
*endp*	distal prong of endopod podomere 7
*en*	endopod(s)
*en1–6*	endopod podomeres 1–6
*ex*	exopod(s)
*exdl*	distal lobe of exopod
*expl*	proximal lobe of exopod
*gl*	glabella
*hy*	hypostome
*il*	inner lamella
*mn?*	possible median node
*mr*	marginal rim
*ms1–11*	pygidial marginal spine
*on*	occipital node
*or*	occipital ring
*OS*	occipital segment (fourth appendage bearing segment
*P1*	first pygidial segment (15th trunk segment/20th appendage bearing segment)
*pa1–3*	postantennular appendages 1–3
*phs*	prehypostomal sclerite
*pc*	preglabellar sagittal crease
*pw*	posterior wings of hypostome
*T1–14*	tergites 1–14 (tergites of fifth to 19th appendage bearing segments)

### Cladistic analysis

The cladistic analysis of 74 characters scored for 54 taxa (section Taxa included in the analysis) was run in TNT [57] under both equal and implied weights. A heuristic search algorithm was employed with 10000 replicates using the ratchet and drift algorithms. Searches were carried out both unweighted and with implied weights using concavity constants [58]*k*=1–20, 22, 24, 26, 28, 30, 35, 40, 50, 75, 100, 150, 300, 400, 500, 750, and 1000. A file including the character matrix and trees obtained under unweighted analysis is included in .nex format within the article’s Additional file 1.

The characters are broadly based on those of J. Ortega Hernández, D. A. Legg, and S. J. Braddy [19], but the taxonomic scope has been altered, and a number of character codings changed. *Shankouia zhenghei* Chen, Wang, Maas and Waloszek in Waloszek, Chen, Maas & Wang, 2005 has been used as an out-group rather than *Fortiforceps foliosa* Hou & Bergström, 1997. The latter has been discussed as a possible derivative of the chelicerate stem-lineage [21,23] and therefore is more suitable as part of the analysed in-group. All aglaspidid taxa except *Aglaspis spinifer* Raasch, 1939 and *Flobertia kochi* Hesselbo, 1992 have been removed since internal aglaspidid relationships are not the focus of the present analysis. The problematic “arachnomorphs” *Nettapezoura basilika* Briggs, Lieberman, Hendricks, Halgedahl & Jarrard, 2008, *Dicranocaris guntherorum* Briggs, Lieberman, Hendricks, Halgedahl & Jarrard, 2008 from the Cambrian Series 3 of Utah, the putative artiopodans *Panlongia* Liu & Luo *in* Liu, Luo, Chen & Hu, 2006, *Buenaspis forteyi* Budd, 1999, and *Pygmaclypeatus daziensis* Zhang, Han & Shu, 2000, and the cheloniellids *Duslia insignis* Jahn, 1893, *Neostrabops martini* Caster & Macke, 1952, *Pseudarthron whittingtoni* Selden & White, 1983, and *Triopus draboviensis* Barrande, 1872 have been removed because of the paucity of information on their morphology. The scope of artiopodan taxa has been extended by *Arthroaspis bergstroemi* n. gen. n. sp., *Australimicola spriggi* Paterson, García-Bellido & Edgecombe 2012, *Campanamuta mantonae* Budd, 2011 and the nektaspids *Emucaris fava* Paterson, Edgecombe, García-Bellido, Jago & Gehling, 2010 and *Kangacaris zhangi* Paterson, Edgecombe, García-Bellido, Jago & Gehling, 2010, and the trilobite *Triarthrus eatoni* (Hall, 1838). Further taxa added are the problematic *Agnostus pisiformis* (Wahlenberg, 1818), *Isoxys volucris* Williams, Siveter & Peel, 1996, and *Kiisortoqia soperi* Stein, 2010, and the stem lineage crustaceans *Henningsmoenicaris scutula* (Walossek & Müller, 1990), *Oelandocaris oelandica* Müller, 1983. The taxa included in the analysis are summarized in the section Taxa included in the analysis.

### **Taxa included in the analysis**

*Shankouia zhenghei Chen, Wang, Maas and Waloszek *in* Waloszek, Chen, Maas & Wang, 2005

*Aglaspis spinifer* Raasch, 1939

*Agnostus pisiformis* (Wahlenberg, 1818)

*Alalcomenaeus cambricus* Simonetta, 1970

*Arthroaspis bergstroemi* n. gen. n. sp.

*Australimicola spriggi* Paterson, Garcíía-Bellido & Edgecombe, 2012

*Burgessia bella* Walcott, 1912

*Campanamuta mantonae* Budd, 2011

*Cheloniellon calmani* Broili, 1932

*Cindarella eucalla* Chen, Ramsköld, Edgecombe & Zhou *in* Chen, Zhou, Zhu & Yeh, 1996

*Emeraldella brocki* Walcott, 1912

*Emucaris fava* Paterson, Edgecombe, García-Bellido, Jago & Gehling, 2010

*Eoredlichia intermedia* (Lu, 1940)

*Flobertia kochi* Hesselbo, 1992

*Fortiforceps foliosa* Hou & Bergström, 1997

*Haikoucaris ercaiensis* Chen, Waloszek & Maas, 2004

*Helmetia expansa* Walcott, 1918

*Henningsmoenicaris scutula* (Walossek & Müüller, 1990)

*Isoxys volucris* Williams, Siveter & Peel, 1996

*Jiangfengia multisegmentalis* Hou, 1987a

*Kangacaris zhangi* Paterson, Edgecombe, García-Bellido, Jago & Gehling, 2010

*Kiisortoqia soperi* Stein, 2010

*Kuamaia lata* Hou, 1987b

*Kwanyinaspis maotianshanensis* Zhang & Shu, 2005

*Marrella splendens* Walcott, 1912

*Martinssonia elongata* Müller & Walossek, 1986

*Mimetaster hexagonalis* Gürich, 1931

*Misszhouia longicaudata* (Zhang & Hou, 1985)

*Molaria spinifera* Walcott, 1912

*Naraoia compacta* Walcott, 1912

*Naraoia spinosa* Zhang & Hou, 1985

*Oelandocaris oelandica* Müller, 1983

*Olenoides serratus* (Rominger, 1887)

*Phytophilaspis pergamena* Ivantsov, 1999

*Retifacies abnormalis* Hou, Chen & Lu, 1989

*Saperion glumaceum* Hou, Ramsköld & Bergström, 1991

*Sidneyia inexpectans* Walcott, 1911

*Sinoburius lunaris* Hou, Ramsköld & Bergström, 1991

*Siriocaris trollae* Lagebro, Stein & Peel, 2009

*Skioldia aldna* Hou & Bergström, 1997

*Soomaspis splendida* Fortey & Theron, 1994

*Squamacula clypeata* Hou & Bergström, 1997

*Tariccoia arrusensis* Hamman, Laske & Pillola, 1990

*Tegopelte gigas* Simonetta & Delle Cave, 1975

*Triarthrus eatoni* (Hall, 1838)

*Weinbergina opitzi* Richter & Richter, 1929

*Xandarella spectaculum* Hou, Ramsköld & Bergström, 1991

*Yohoia tenuis* Walcott, 1912

Eurypterida

*Leanchoilia superlata* Walcott, 1912 and *Leanchoilia illecebrosa* (Hou, 1987) (combined)

Nebalia

*Liwia plana* (Lendzion, 1975) and *convexa* (Lendzion, 1975) (combined)

Pycnogonida

### Characters

Most of the characters are derived, with modification, from Ortega Hernández, Legg, and Braddy [19]. Characters pertinent only to the internal relationships of aglaspidids have been removed, whilst other characters have been added from previous studies of Cambrian arthropods [31,32,59,60]. The characters are listed below. Explanations are given where they have been significantly modified or the scoring differs from that of previous authors.

1. Nature of first cephalic appendage: (0) limb-like appendage; (1) antennae. Modified from Ortega Hernández *et al*. character 1. Limb-like appendage, substituted for raptorial [19], applies to any appendage that is not a specialised sensorial appendage. This character was coded by Ortega Hernández *et al*. as state one for *Aglaspis*, *Kwanyinaspis*, and *Sinoburius*, but has been coded as uncertain for all of these taxa here. No antennulae are preserved in *Kwanyinaspis* and from *Aglaspis* only the most proximal articles are known [61] while in *Sinoburius* there are only impressions that have been interpreted as antennulae [12,26]. The antennula of *Martinssonia* is interpreted as a feeding antennula [9] as is that of *Rehbachiella*[10].

2. Composition of first cephalic appendage: (0) 7–15 articles; (1) > 15 articles; (2) ≤ 7 articles. Modified from Stein and Selden character 19 [31], the autapomorphic state for *Oelandocaris* has been removed and the character is here coded as unknown for that taxon. Contrary to Stein and Selden, the character is not coded as state 0 for *Saperion*; the antennula in the specimen on which that coding was based (fig. 3.1, 7.1 of Edgecombe and Ramsköld [26]) appears to be incomplete, preservation terminates at a scarp.

3. Armature of first cephalic appendage: (0) none; (1) pair of simple, robust spines medioventrally on each article; (2) setae on mediodistal margin of articles; (3) single, large, medodistal spine or finger. Modified from Stein and Selden character 21; the autapomorphic state for *Oelandocaris* has been removed and the character is coded as unknown for that taxon.

4. Number of articles with fingers in short feeding appendage: (0) four; (1) three; (2) two. Modified from Ortega Hernández *et al*. character 2. The character is here coded unknown for *Aglaspis*, *Kwanyinaspis*, and *Sinoburius*, rather than inapplicable because the antennular morphology is not known for these taxa. The character was coded as state 2 for *Fortiforceps* and state 1 for *Yohoia* by Ortega Hernández *et al*., but both have four fingers [23].

5. Long spinose projections on distal part of terminal three podomeres of great appendage bearing a flagellum: (0) absent; (1) present. Substituted for character 4 of Ortega Hernández *et al*. Modified from character 6 of Edgecombe *et al*. [59]. Here coded unknown for *Aglaspis*, *Kwanyinaspis*, and *Sinoburius*, rather than inapplicable as coded by Ortega Hernáández *et al*.

6. Feeding appendage with elbow joint: (0) absent; (1) present. This is a newly added character based on the recognition of an elbow joint in the great appendage of megacheirans [23].

7. Number of appendage bearing post-ocular segments incorporated into the head/prosoma: (0) one; (1) four; (2) five; (3) six; (4) seven. Modified from character 5 of Ortega Hernández *et al*. *Cheloniellon* has been coded as having four post-ocular segments (state 1) rather than five. Figure 11a in the redescription by W. Stürmer and J. Bergström [62] shows that the fourth appendage posterior to the antennula inserts under the first trunk tergite, the fifth under the second. Then there is a gap due to the appendages posteriorly being flipped backward. *Marrella* and *Mimetaster* are coded as unknown. Counts of segments in these taxa seem to rest on the number of specialised appendages, while in other taxa the number of segments carrying unspecialised appendages incorporated into the cephalic shield is counted. In both taxa, the latter count can not be established. The character is coded unknown for *Naraoia compacta*. The character is coded as state 3 (six segments) for Pycnogonida rather than state 4 (seven segments). It is unknown for *Sidneyia* and *Squamacula*; there is no direct evidence for cephalic appendages available in *Squamacula*, and reexamination of the *Sidneyia* material revealed the presence of gnathobases in the head, though the number cannot be established (Stein in preparation). The number is unknown for *Sinoburius*, where only impressions of the appendages are known.

8. Exopod on first post-antennular appendage: (0) present; (1) absent. Modified from character 6 of Ortega Hernández *et al*. This was coded as state 1 for *Emeraldella*; the character is coded here as unknown for that taxon since, while evidence for presence of an exopod in that limb is inconclusive, there is no evidence of absence [31]. The character has been coded as unknown for *Liwia*, as no postantennular appendages are preserved. Likewise in *Sinoburius* where only impressions of the appendages are preserved with no evidence of exopods and *Sidneyia*, *Squamacula*, *Skioldia*, and *Saperion* where number and morphology of the cephalic appendages are unknown. In *Marrella*, the second cephalic appendage is a swimming arm. A similar swimming arm occurs in the marrellomorph *Vachonisia rogeri* Lehmann, 1955 [63] where it is formed by the exopod, and given the correspondence in morphology, this appendage in *Marrella* is here interpreted as an exopod. The character is coded as unknown for *Mimetaster*.

9. Exopod on second post-antennular appendage: (0) present; (1) absent. Character 7 of Ortega Hernández *et al*. The character has been coded as unknown for *Liwia*, *Saperion*, *Sidneyia*, *Sinoburius*, *Skioldia*, and *Squamacula* (see comment on previous character). The second post-antennular appendage of *Marrella* carries an exopod independent of whether or not it is incorporated into the head, and it has been coded as state 0. The second postantennular appendage of *Mimetaster* is uniramous [64] but by comparison with *Vachonisia*, where the main rami are the exopods [63], it is not clear if the rami in *Mimetaster* are endopods or exopods, and the character has been coded as unknown for that taxon.

10. Insertion of cephalic exopods: (0) as in trunk; (1) shorter joint. Character 31 of Stein and Selden.

11. Cephalic endopods: (0) as in trunk limbs; (1) first postantennular limb with fewer podomeres or reduced; (2) endopod of first postantennular limb heavily reduced or absent, of second postantennular limb considerably more slender than in trunk and fewer podomeres. Modified from character 33 of Stein & Selden. Coded as inapplicable for Pycnogonida where the second cephalic appendage is the oviger, and Eurypterida where all appendages are differentiated.

12. Composition of first and second cephalic exopods: (0) as in trunk limbs; (1) multiarticulate, each article with mediodistal setae or pair of lateral setae. Character 34 of Stein and Selden.

13. Trunk endopods: (0) absent or reduced; (1) present. Character 9 of Ortega Hernández *et al*. Coded inapplicable for Pycnogonida where there is no trunk. No postantennular appendages are preserved in *Liwia*, so the character is coded as unknown for that taxon.

14. Exopod structure: (0) simple oval flap; (1) bilobate flap, exopod differentiated into proximal and distal lobes; (2) numerous articles; (3) book gills. Modified from character 10 of Ortega Hernández *et al*. Specifics on setation have been removed from this character. State 3 of Ortega Hernández *et al*. (undivided with lamellar setae) which was autapomorphic for *Retifacies*, has been removed since the exopod structure of that taxon is not well known. It is unclear if the most distal lamella as drawn in figure 51 of Hou and Bergström [12] is a lamella or the distal article of a bilobate exopod. The character has been coded unknown for *Retifacies*, and further for *Burgessia*, for which details of the exopods are also unknown. It is coded as unknown for *Liwia*, *Sinoburius*, and *Skioldia*. For *Skioldia* only impressions of the lamellae are known (figure 16.54 in [65]). *Nebalia* has differentiated exopods in the cephalic appendages, but the exopods of the more serially similar thoracopods and pleopods are best described as simple flaps. Similarly, in *Martinssonia* and *Rehbachiella* the exopods of the more posterior appendages are simple flaps [7,10]. Those of *Sidneyia* are differentiated into proximal and distal lobes, with the proximal lobe carrying the lamellae (Stein in preparation). In *Squamacula* only the large flap is visible [66], but if the appendage structure is similar to that of *Sidneyia*, the proximal lobe could be inaccessible and the character is coded as unknown for that taxon. The exopods of *Tegopelte* are differentiated into proximal and distal lobes as best seen in newly published photographs [67]. There is also evidence of the exopods in *Cheloniellon* being differentiated into proximal and distal lobes; the flap-like lobe clearly inserts into a narrower portion in figures 8a, 11 of Stürmer and Bergström [62].

15. Proximal lobe of exopod: (0) flattened lobe; (1) slender shaft. Character 11 of Ortega Hernández *et al*. Coded unknown for *Burgessia*, *Cheloniellon*, and *Squamacula* (see discussions in previous character). *Tegopelte* is coded as state 0. The proximal lobe of the exopods in *Sidneyia* is also a flattened lobe (state 0).

16. Distal lobe of exopod: (0) small to moderate sized flap; (1) large, teardrop shaped lobe with long attachment; (2) dominant part of appendage, shielding endopod. Modified from character 12 of Ortega Hernández *et al*. Unknown for *Burgessia* and *Cheloniellon* (see previous two characters). The distal lobes of *Kwanyinaspis*[68] and *Naraoia spinosa*[69] are relative to the proximal lobe better characterised by state 1, cf. *Kuamaia*[12]. *Tegopelte* has a moderate sized flap [67]. State 2 has been introduced to describe the condition in *Sidneyia* (Stein in preparation). *Squamacula* is coded for that state because of the characteristic shielding of the endopod.

17. Joints in flap-like exopod (0) absent; (1) one; (2) two. Modified from character 13 of Ortega Hernández *et al*. The term joint is used rather than septum. The character is coded as unknown for *Burgessia* and *Cheloniellon* (see characters 14–16). Character state 2 has been introduced for the state in *Emeraldella*[31] and *Sidneyia* (Stein in preparation). The distal lobe in the exopod of *Kwanyinaspis* is clearly set off by a joint, extending from the basipod/exopod joint at a slight angle to the lateral margin where the lamellar insertion ends and the distal lobe starts (figure 2D in [68]). The character is coded unknown for *Martinssonia*, where there could be a single joint (figure 10A in [7]), but evidence is inconclusive.

18. Imbricate exopod lamellae: (0) absent; (1) present. Character 14 of Ortega Hernández *et al*. Coded unknown for *Burgessia* and *Cheloniellon* (see characters 14–17). The character is also unknown for *Liwia*, of which no postantennular appendages are preserved, and *Sinoburius*, for which exopod preservation is poor (figure 77A in [12]). If *Squamacula* had a short proximal lobe like *Sidneyia*, lamellae could be present but not preserved, so the character has been coded as unknown for that taxon as well. Lamellae are present in *Tegopelte*[67] and *Marrella*[70]. It is unclear if the filamentous setae of *Mimetaster* are lamellae [64].

19. Non-overlapping marginal setae: (0) absent; (1) small setae; (2) long spines. Modified from character 16 of Ortega Hernández *et al*. Coded as unknown for *Burgessia* (see characters 14–18) and for *Kuamaia* and *Saperion* where setae may be absent due to preservation of the few specimens that preserve the distal lobe [12,26]. The character is coded unknown for *Haikoucaris*, where preservation of the exopods does not allow assessment of whether the preserved structures are lamellae or setae. Unknown also for *Retifacies* (see character 14) and *Mimetaster* (see character 18). Pycnogonida do not have exopods, so the character has been coded as inapplicable rather than absent. The large distal lobe of *Sidneyia* is fringed by very fine setae (picture) and small setae are also found on the pleopods of *Nebalia* and posterior exopods of *Rehbachiella*. *Martinssonia* has long (relative to the body size) spines. Ortega Hernández *et al*. coded the character as inapplicable for *Martinssonia*, *Rehbachiella*, and *Nebalia* because of their exopod shape, but in all three taxa, the more posterior appendage have paddle shaped (oval) exopods (see character 14) and are here coded. The ‘teeth’ in *Weinbergina*[71] are here interpreted as setae.

20. Insertion of exopod on trunk appendages: (0) along lateral edge of multiple podomeres of limb axis; (1) on basipod and first endopod podomere; (2) on basipod only. Character 30 of Stein and Selden.

21. Setae of anterior exopods facing endopods: (0) absent; (1) present. New character. This is present in *Oelandocaris*, *Henningsmoenicaris*, *Martinssonia*, and *Rehbachiella*, where exopod setation is mainly on the median side of the anterior cephalic exopods.

22. Body–limb joint: (0) short, sclerotized, pivot-jointed rings; (1) arthrodial membrane with partially sclerotized half-rings; (2) arthrodial membrane. Character 23 of Stein and Selden.

23. Endites on basipod/gnathobase: (0) absent; (1) multiple spinose endites; (2) single spinose endite. Modified from character 24 of Stein and Selden. Here coded as unknown for *Saperion*, as the proximal portions of the appendages of that taxon are not well known.

24. Composition of endopod: (0) nine or eight podomeres; (1) seven podomeres; (2) fewer than seven podomeres. Modified from character 25 of Stein and Selden. According to [24] the endopod of *Leanchoilia superlata* consists of only 7 podomeres, but an isolated limb figured by D. L. Bruton and H. B. Whittington in their figure 105 clearly shows 9 [72].

25. Median armature of podomeres: (0) none; (1) mediodistal spines or spinules/denticles on all but penultimate and distal podomeres; (2) biserial spines or spinose endites along median edge of podomeres 1–4. Character 27 of Stein and Selden.

26. Proximal endite or coxa as separate sclerotized element proximal to limb base in cephalic appendages (0) absent; (1) present. New character, derived from the analysis of J. T. Haug, A. Maas, and D. Waloszek (there coded individually for appendages and ontogenetic stages, characters 6, 7, 13, 14, 20, 21) [60]. Here, only the appearance of a proximal endite on any limb is coded.

27. Position of lateral eyes: (0) ventral; (1) dorsal. Modified from character 18 of Ortega Hernández *et al*., coded as state 0 where eyes are not dorsal, even if they are not preserved. An exception is *Weinbergina*, where eyes are not preserved, but ophthalmic ridges indicate a dorsal position. For *Pycnogonida* and *Martinssonia*, where there is positive evidence for the absence of lateral eyes (the eyes of pycnogonids are interpreted as median eyes [73]), this is coded as inapplicable. The putative dorsal eyes of *Cheloniellon*[62] could be the large, dorsoventrally extending gnathobases of the anterior trunk appendages superimposed on the head shield in the radiographs. The character is coded here as unknown for that taxon. It is coded unknown for *Liwia*, where the dorsal cuticle of the cephalon is not preserved [74] and *Marrella*, where position and preservation of the eyes is unclear. By comparison with the position and structure of the eyes in *Henningsmoenicaris*[60,75] the eyes of *Oelandocaris* are interpreted as lateral eyes and are ventral.

28. Visual surface with calcified lenses, bounded with circumocular suture: (0) absent; (1) present. Character 21 of Ortega Hernández *et al*.

29. Dorsal bulge in exoskeleton accommodating drop-shaped ventral eyes: (0) absent; (1) present. Character 22 of Ortega Hernández *et al*. The eyes of helmetiids and *Kwanyinaspis* appear to be in sharply defined bulges (e.g. figure 5 in [26]), which does not seem to be the case in *Sinoburius* where the bulges are confluent with the shield (figure 77B–D in [12]) and could be compactional artefacts. The character is coded as unknown for that taxon.

30. Eye slits: (0) absent; (1) present. Character 23 of Ortega Hernández *et al*. This is often interpreted as present in *Sinoburius*[26], but the evidence is inconclusive and the character is here coded as unknown for that taxon.

31. Dorsal median eyes: (0) absent; (1) present. Character 24 of Ortega Hernández *et al*.

32. Bilobate lateral eyes where four visual surfaces are arranged in a subtransverse band across head shield: (0) absent; (1) present. Modified from character 9 of Edgecombe *et al*., taking a recent reinterpretation of the eyes of *Leanchoilia superlata* into account [24].

33. Expanded cephalic doublure: (0) absent; (1) concentric; (2) crescentic. Modified from character 27 of Ortega Hernández *et al*. State 2 occurs in *Soomaspis* and *Tarricoia*. Contrary to the coding of Ortega Hernández *et al*., the cephalic doublure is unknown for *Burgessia*, *Haikoucaris*, *Retifacies*, and *Tegopelte*. An expanded doublure is absent in *Cheloniellon* where doublure covers less than 10% of the total width of the head (figure 12C in [62]), likewise in *Cindarella* (figure 5g in [76]), *Xandarella* (figure 68C, 70 in [12]), *Emeraldella*[31]*Misszhouia*[77], *Naraoia compacta* (pl. 10:9 in [78]) and *N*. *spinosa*[77], and *Sinoburius* (figure 1 in [26]). If the interpretation of the elevated structure anteromedially in the cephalon of *Kwanyinaspis* as the hypostome (figure 1b in [19]) is correct, the taxon can not have an expanded doublure, since the structure extends all the way to the very margin. Accordingly, the character is coded as state 0 for this taxon. The character is also coded as unknown for *Squamacula* where often a doublure covering the whole ventral side of the cephalon is inferred, as in *Sidneyia*. In *Sidneyia*, this is an artefact of folding under of the anterior margin of the cephalon (Stein in preparation).

34. Anteromedian margin of cephalon notched, accommodating strongly sclerotised plate: (0) absent; (1) present. Character 28 of Ortega Hernández *et al*.

35. Hypostomal sclerite: (0) wide attachment with or without suture; (1) natant; (2) with narrow overlap with prehypostomal sclerite; (3) absent. Modified from character 29 of Ortega Hernández *et al*.; their states 0 and 3 have been merged. The character is coded unknown for *Australimicola* (contra [32]) where the mode of attachment can not be confirmed in the available photographs. It is also coded unknown for *Burgessia* where the presence and state of the hypostome are entirely unclear. The same is true for *Fortiforceps*, *Haikoucaris*, *Jianfengia*, *Yohoia*, *Sinoburius*, and *Tegopelte*. There is sclerotized cuticle covering the backward curved mouth in *Alalcomenaeus* (e.g. pl. 6:5, 3 and text-figures 10–11 in [79]) which represents the hypostome, and the attachment has to be natant because of the eyes between the hypostome and the anterior margin of the cephalon. The same is true for *Leanchoilia*[24,80]. The hypostome of *Martinssonia* is incorporated into the ‘forehead’ and its delimitation from the remainder of that and relation with the head shield margin is unclear, and the character is coded as unknown for *Martinssonia*. In *Nebalia* and *Rehbachiella* the hypostome is subsumed by the labrum, but the whole hypostome-labrum complex is natant.

36. Hypostome divided into anterior and posterior parts by transverse suture: (0) absent; (1) present. Character 13 of Paterson *et al*. [32,81]. This is coded as unknown for *Marrella* where the hypostome morphology is unclear.

37. Labrum: (0) absent; (1) present. Character 1 of Haug *et al*. [60].

38. Prehypostomal sclerite: (0) as individual sclerite anterior to head tergite or recessed in notch in head shield; (1) incorporated in doublure as rostral plate or reduced. Character 5 of Stein and Selden, substituted for character 30 of Ortega Hernández *et al*. Coded as unknown for *Yohoia*[23].

39. Frontal organs on prehypostomal sclerite: (0) absent; (1) present. Character 11 of [32,81]. It is questionable whether the spots observed on the prehypostomal sclerite in *Helmetia*[26] are homologous to the bulges in nektaspids as they differ in preservation, which is more like that of internal structures preserved more posteriorly in USNM 83952. The character is here coded as absent for *Helmetia*.

40. Visible ecdysial dorsal sutures: (0) absent; (1) present. Character 31 of Ortega Hernández *et al*.

41. Position of ecdysial sutures: (0) marginal; (1) dorsal. Character 32 of Ortega Hernández *et al*.

42. Elevated marginal rim: (0) absent; (1) present. Character 33 Ortega Hernández *et al*. There is no elevated marginal rim in *Kwanyinaspis*, contrary to the coding of Ortega Hernández *et al*.

43. Distinct trilobation of head shield, marked by axial furrows: (0) absent; (1) present. Character 6 of Stein and Selden. This is unclear for *Siriocaris* and coded unknown for the taxon.

44. Dorsal expression of last segment of head shield: (0) absent; (1) present. Character 7 of Stein and Selden.

45. Head shield outline: (0) genal angles, rounded, acute or with spines; (1) lateral spine-like extensions of the head shield; (2) lateral margin extending into genal spines flanking anterior trunk. Modified from character 36 of Ortega Hernández *et al*. Their states 0, 1 and 2 have been collapsed into a single state. State 2 is new and occurs in aglaspidids, trilobites, and *Sinoburius*. The character has been coded as state 0 for *Saperion* and *Skioldia*, where the posterolateral corners of the cephalon are visible and their morphology can be described, even if it is debated whether the articulation between cephalon and trunk tergites is functional.

46. Mineralized cuticle: (0) absent; (1) calcitic; (2) phosphatic. Modified from character 40 of Ortega Hernández *et al*. (see also Paterson *et al*. [32]).

47. Trunk tergites with expanded tergopleurae covering appendages dorsally: (0) absent; (1) present. Character 41 of Ortega Hernández *et al*. (cf *pars* character 10 of Stein and Selden). Coded inapplicable for Pycnogonida which does not have a trunk.

48. Free thoracic tergites: (0) absent; (1) present. Character 42 of Ortega Hernández *et al*. Coded inapplicable for Pycnogonida which does not have a trunk.

49. Decoupling of thoracic tergites and segments: (0) absent; (1) present. Character 43 of Ortega Hernández *et al*. Coded inapplicable for Pycnogonida which does not have a trunk.

50. Tergite articulations: (0) tergites non-overlapping, edges separated by arthrodial membrane; (1) extensive overlap of tergites; (2) edge-to-edge pleural articulations. Character 44 of Ortega Hernández *et al*. Coded unknown for *Burgessia*, where little of the tergites is known, and *Sinoburius*, where preservation of the minute specimens is too coarse to discern tergite articulations. Articulations are equally unknown for *Marrella* and *Mimetaster*. *Australimicola* has been coded as having overlapping tergites rather than edge to edge articulations as coded in the analysis included with the original description [32]. The overlap is shown in figures 10 and 12 of Paterson *et al*. [32] and even labeled as such in their interpretative line drawing (figure 9 in [32]) and overlap is given as up to one half the tergal length in the description [32]. *Kangacaris* on the other hand seems to have edge to edge articulations (plate 5 of [81]) while articulations are unclear for *Emucaris*. *Soomaspis*, too, has edge to edge articulating tergopleurae (pl. 1:3 in [82]). The character is coded for *Skioldia*, where articulations are clearly visible and are edge to edge (figure 16.54 in [65]). Coded inapplicable for Pycnogonida which does not have a trunk.

51. Cephalic articulation fused: (0) absent; (1) present. Character 46 of Ortega Hernández *et al*. The authors stated that this was present in *Skioldia*[19] but coded it as absent, which is probably correct. Coded inapplicable for Pycnogonida which does not have a trunk.

52. Head shield overlap of thoracic tergites: (0) overlap absent or identical to overlap between thoracic segments; (1) head shield covers first thoracic tergite only; (2) head shield covers multiple anterior trunk tergites. Character 47 of Ortega Hernández *et al*. This character is coded unknown for *Marrella* and *Mimetaster*, where the posterior boundary of the head shield is unclear. Overlap in *Martinssonia* is clearly identical to the trunk tergites [7]; there is no overlap of multiple segments as coded by Ortega Hernández *et al*.. The same is the case in *Sinoburius*. Despite the possibly non-functional articulations, overlap between the head shield and the first tergite is identical to that between subsequent tergites in *Saperion* and *Skioldia* and can be coded. Coded inapplicable for Pycnogonida which does not have a trunk.

53. Trunk narrowed anteriorly relative to head shield, widest posteriorly: (0) absent; (1) present. Character 49 of Ortega Hernández *et al*. Coded inapplicable for Pycnogonida which does not have a trunk.

54. Raised axial region of trunk defined by axial furrows: (0) absent; (1) present. Modified from character 54 of Ortega Hernández *et al*. (cf pars character 10 of Stein and Selden). The character has been coded absent for *Kuamaia*, where the axis in the fossils is defined by compactional folds as in *Arthroaspis*. The axis of *Weinbergina* is defined by nodes rather than furrows as is best seen in the non-lateral compressed specimens (figure 6a in [83]). Coded inapplicable for Pycnogonida which does not have a trunk.

55. Articulating device: (0) articulating half-ring and flanges; (1) articulating ridge/anterior process; (2) no skeletal device. Modified from character 55 of Ortega Hernández *et al*. (anterior tergal process absent/present). Coded unknown for *Alalcomenaeus*, *Burgessia*, *Cindarella*, *Fortiforceps*, *Haikoucaris*, *Jianfengia*, *Marrella*, *Mimetaster*, *Sinoburius*, *Squamacula*, *Tariccoia*, *Xandarella*, and *Yohoia*. Articulating ridges are present in *Cheloniellon* (figure 12a in [62]), *Leanchoilia*[72], *Retifacies*[12], *Sidneyia* (pl. 5:37 in [84]), Eurypterida, and *Weinbergina* (figure 4c in [83]). The articulating half-ring (state 0) is visible in the holotype of *Liwia convexa* (figure 4D in [74]) and is also present in *Soomaspis* (text-figure 1b–c in [82]). No skeletal articulation devices are present in *Martinssonia*, *Rehbachiella*, *Nebalia*, and the pycnogonids. The character is coded inapplicable for taxa without free tergites (*Misszhouia* and *Naraoia*) or where the tergites are fully effaced (*Tegopelte*). Coded inapplicable for Pycnogonida which does not have a trunk with articulating tergites.

56. Postcephalic tagmosis: (0) uniform trunk with free tergites and telson or pygidium; (1) bipartite trunk with free tergites and telson, segments in abdomen apodous, tergites ring-shaped, without tergopleurae. Modified from character 2 of Stein and Selden. The pygidium is here defined as a uniform shield formed by the ankylosed posterior tergites, and not considered a true tagma [85]. Aglaspidids as *Emeraldella* are not considered to have an abdomen. Tergopleurae are present, though posterior limbs are never known. This is unclear for *Cheloniellon* where the tergites of the posterior most segments appear to lack tergopleurae, but seem to be appendage bearing. The posterior end of *Flobertia* is poorly known and the character is coded as unknown also for that taxon. Coded inapplicable for Pycnogonida which does not have a trunk.

57. Trunk segments form single shield: (0) absent; (1) present. Character 3 of Stein and Selden. Coded inapplicable for Pycnogonida which does not have a trunk.

58. Posterior tergites strongly curved in dorsal aspect compared to anterior tergites: (0) absent; (1) present. Modified from character 61 of Ortega Hernández *et al*. Here coded inapplicable where posterior segments are incorporated into a large pygidium or no tergopleurae are present. This is further coded absent for all taxa where only the tergopleural extremity curves. Unknown for *Burgessia*, *Marrella*, and *Mimetaster* where little is known of tergite morphology. Coded inapplicable for Pycnogonida which does not have a trunk.

59. Pygidium: (0) absent; (1) present. Character 62 of Ortega Hernández *et al*. Coded as unknown for *Cindarella*, *Henningsmoenicaris*, *Squamacula*, and *Xandarella*. Evidence for a pygidium in *Squamacula* and *Australimicola* is inconclusive. In *Cindarella* and *Xandarella* a number of tergites cover an increasing number of appendiferous segments posteriorly. This seems incompatible with recent interpretations of the pygidium as a frozen growth zone [85]. Further, a recently described xandarellid, *Luohuilinella rarus* Zhang, Fu & Dai, 2012, appears to be lacking a pygidium or elongate posterior tergites [86]. The absence of a pygidium in a member of Xandarellida casts further doubt on the nature of the other xandarellids’ elongate tergites as a true pygidium. *Henningsmoenicaris scutula* has been described as having a developmental mode where segments are released from a growth zone, but until release aggregate in that growth zone [60]. This is technically a pygidium, but since the known stages of *Henningsmoenicaris* are immature and it is unclear if a pygidium is retained in the adult, the character is scored as unknown. Coded inapplicable for Pycnogonida which does not have a trunk.

60. Position of the anus: (0) terminal; (1) ventral. Character 63 of Ortega Hernández *et al*. Coded unknown for *Australimicola*, *Marrella*, and *Mimetaster*. The position of the anus is not preserved in *Aglaspis*, but because the styliform telson is closed posteriorly, it is likely to have been at the base of the telson. The same in *Kwanyinaspis*. The character is coded inapplicable for pycnogonids, where the telson is reduced.

61. Pygidium with median keel: (0) absent; (1) present. Character 64 of Ortega Hernández *et al*. Coded unknown for all taxa where the presence of a pygidium is unclear.

62. Pygidium with broad-based median spine: (0) absent; (1) present. Character 65 of Ortega Hernández *et al*. Coded unknown for all taxa where the presence of a pygidium is unclear.

63. Pygidium with lateral spines: (0) absent; (1) present. Character 66 of Ortega Hernández *et al*. Coded unknown for all taxa where the presence of a pygidium is unclear. This is here coded only where the spines are segmental, i.e. remnants of the teopleural tips of the ankylosed tergites. Defined as such, they are absent in *Retifacies*, but present in *Soomaspis* and *Tariccoia*.

64. Size of caudal end: (0) shorter to slightly longer than cephalon; (1) much longer than cephalon. Modified from character 23 of Paterson *et al*. [32,81]. The term caudal end applies to both pygidium and the axial portion of the telson. Coded even for taxa where the presence of a pygidium is unclear. Coded present for the naraoiids, where the whole trunk is considered subsumed by the pygidium which is longer than the cephalon, but absent in *Saperion* and *Skioldia*, where the pygidium is visible despite possibly non-functional articulations, and not much longer than the cephalon. Coded present in *Liwia*, *Soomaspis*, and *Tariccoia*.

65. Free telson: (0) present; (1) absent. Modified form character 67 of Ortega Hernández *et al*. Coded unknown for *Australimicola* where the nature of the caudal region is unclear.

66. Telson shape: (0) styliform keeled; (1) plate-like; (2) tubular cap-like to tubular styliform. Modified from character 68 of Ortega Hernández *et al*. The states have been modified. The telson of *Weinbergina* and Eurypterida is a stylus with a triangilar cross section (keeled), whereas those of *Aglaspis* and *Kwanyinaspis* have more of an extended tubular shape, as also seen, albeit shorter, in *Oelandocaris* and *Henningsmoenicaris*. The telson is seen in the latter two taxa, even though during ontogeny it may be part of a pygidium. Coded unknown for *Australimicola* and other taxa for which the nature of the caudal portion is unclear. Also, the telson shape of *Jianfengia* is unknown, while the character is coded inapplicable for Pycnogonida where the telson is reduced. The megacheirans *Fortiforceps*, *Haikoucaris*, *Leanchoilia*, and *Yohoia* have all been coded as ‘spinose’ (styliform) by Ortega Hernández *et al*., but their morphology is better described as plate-like.

67. Telson carrying furca: (0) absent; (1) present. Newly added character. Coded inapplicable for taxa with a pygidium, unknown for taxa where the nature of the caudal end is unclear.

68. Spines on telson: (0) absent; (1) present. Character 71 of Ortega Hernández *et al*. Coded unknown where the nature of the caudal end is unknown. Furthermore unknown in the megacheirans *Fortiforceps* and *Jianfengia*. Inapplicable for Pycnogonida where the telson is reduced. The telson of *Yohoia* carries spines distally [23].

69. Segmentation of telson: (0) absent (unjointed); (1) present. Character 28 of Paterson *et al*. [32,81]. Coded unknown where the nature of the caudal end is unknown.

70. Postventral plates: (0) absent; (1) present. Character 74 of Ortega Hernández *et al*.

71. Paired modified appendages attached to a differentiated pre-telsonic segment: (0) absent; (1) present. Character 75 of Ortega Hernández *et al*. Filiform caudal cerci are present in *Olenoides*[87], even though they were coded as absent by Ortega Hernández *et al*.

72. Nature of pre-terminal appendages: (0) paddle; (1) cerci. Modified from Ortega Hernández *et al*. character 76; more neutral descriptive terms are adopted here. *Olenoides* has state 1 [87].

73. Relative length of thorax: (0) shorter than caudal end; (1) longer than caudal end. Modified from character 79 of Ortega Hernández *et al*.

74. Differentiation of cephalic gut diverticulae: (0) small, serially regular diverticulae; (1) anterior pair of diverticulae large, expanding across genal region of cephalic shield, with complicated ramification. Character 37 of Paterson *et al*. [32,81]. This is coded absent for *Burgessia* where it is not the anterior pair that is differentiated.

## Results

### Systematic palaeontology

*Arthroaspis* n.g.

### Etymology

*Arthro* from *arthron*―articulation or joint, *aspis*―shield.

### Type species

*Arthroaspis bergstroemi*, by monotypy.

### Diagnosis

Large ‘artiopodan’ attaining maximum length of up to 215 mm with helmetiid-like tergum of edge-to-edge articulating tergites and pygidium with segmental marginal spines. Distinguished from Helmetiida by semi-elliptical cephalon with integral margin lacking recess accommodating prehypostomal sclerite. Cephalon broadly nektaspid-like with preglabellear sagittal crease and natant complex of hypostome and arcuate prehypostomal sclerite. Differentiated from Nektaspida by raised glabella laterally marked by axial furrows and dorsally demarcated occipital segment with occipital ring; trunk differentiated dorsally into thorax of 14 tergites, and pygidium.

### Discussion

*Arthroaspis* n.g. matches the revised diagnosis for Artiopoda [31], but the present analysis does not find support for a monophyletic Artiopoda.

*Arthroaspis bergstroemi* n.sp.

1987 arthropod―S. Conway Morris, J. S. Peel, A. K. Higgins, N. J. Soper & N. C. Davis, p. 182, figure 2g (MGUH 17516) [33]; *non* figure 2a (MGUH 17512), the specimen figured here is *Campanamuta*, see synonymy list in [52].

1996 animal reported as a tegopeltid―L. Ramsköld, J-y. Chen, G. D. Edgecombe, and G-q. Zhou, p. 15 [88].

2007 undescribed arthropod―L. E. Babcock and J. S. Peel, figure 3E (MGUH 28755) [36].

2009 Outer limb branch of an undescribed Sirius Passet (Lower Cambrian of Greenland) lamellipedian arthropod―G. E. Budd and M. J. Telford, p. 815, figure 3f [89].

2010 undescribed arthropod―J. S. Peel, p. 386, figure 2 (MGUH 28755) [39].

2011 *Campanamuta*―G. E. Budd, p. 222 in synonymy list [52].

2011 arthropod—J. S. Peel & J. R. Ineson a figure 4I [37].

2012 “undetermined nonbiomineralized - *Tegopelte*-like arthropods.”—M. G. Mangano, R. G. Bromley, D. A. T. Harper, A. T. Nielsen, M. P. Smith, and J. Vinther, p. 520, figure 1 and in text [54].

### Etymology

In memory of the late Jan Bergström in the 40th year since his publication of *Organization, life, and systematics of trilobites*[90].

### Holotype

MGUH 30382, part and counterpart.

### Other material

MGUH 30383–30419.

### Type locality and horizon

*Arthroaspis bergstroemi* is one of the most common elements within the Sirius Passet Lagerstätte occurring in various lithologies both within the main Sirius Passet locality and at localities 2 and 3 described by J. S. Peel and J. R. Ineson [37]. Cambrian, Series 2, Montezuman Stage.

### Diagnosis

As for genus.

### Description

**Figure 1:**
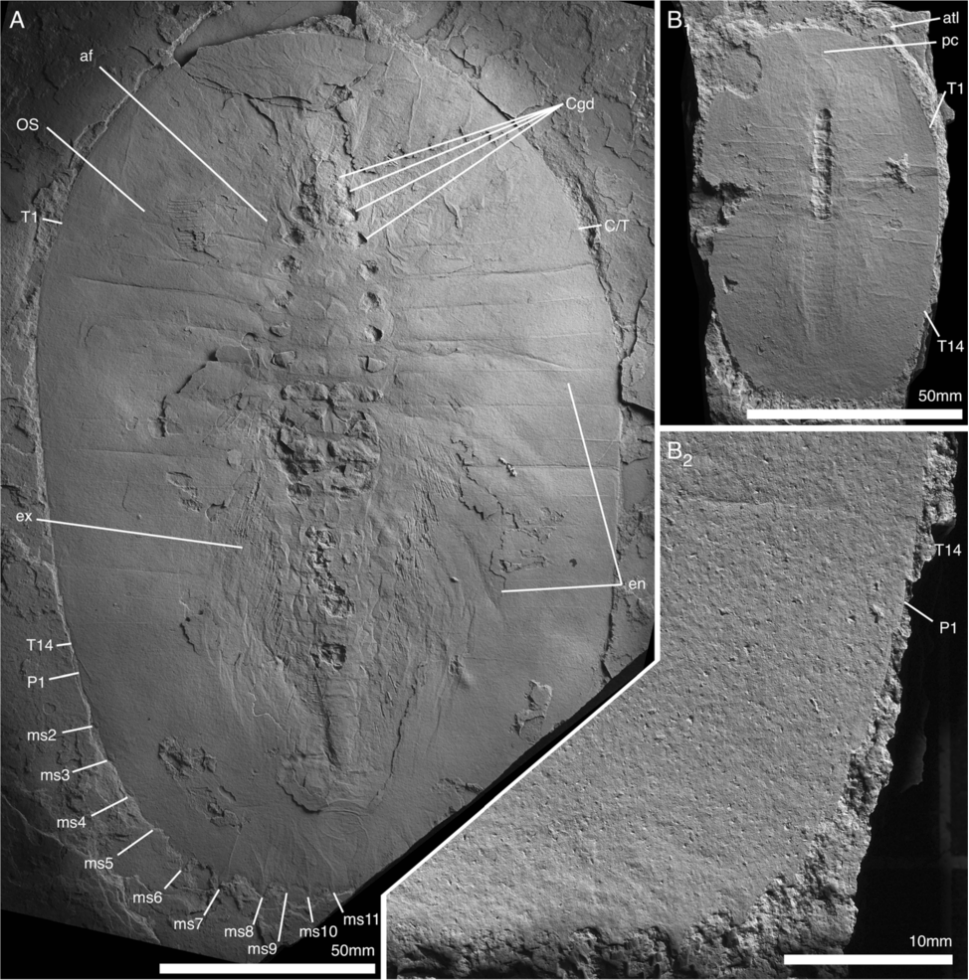
***Arthroaspis bergstroemi *n. gen. n. sp. A**. MGUH 30382pt, holotype, large individual (200 mm); **B**. MGUH 30405, small individual (87 mm), **B**_**1**_ same scale as **A**. Both specimens with 14 thoracic tergites, indicating that the small specimen already is a holaspid. For abbreviations see Table 1.

**Figure 2:**
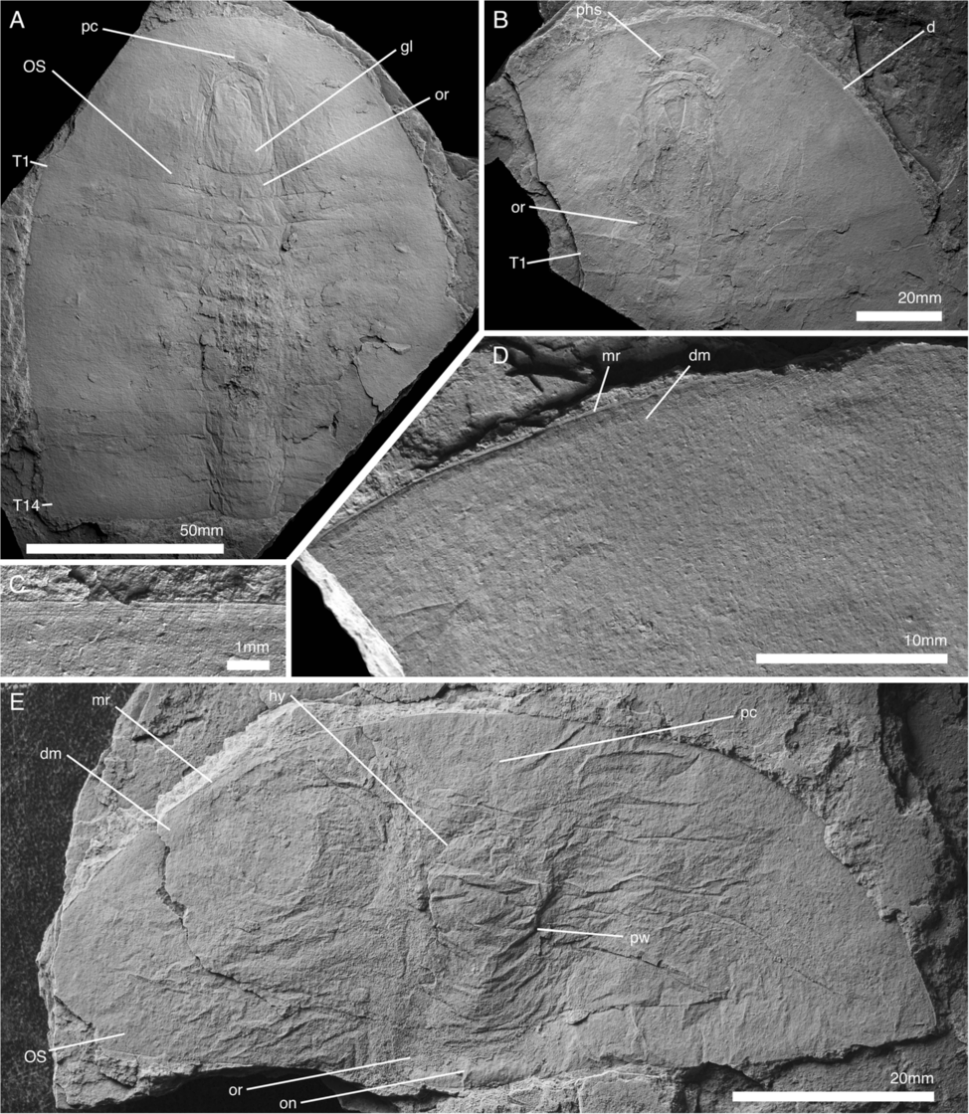
**Cephalon and anterior trunk of *Arthroaspis bergstroemi *n. gen. n. sp. A**. MGUH 30411 specimen with cephalon and fully articulated thorax. Potential muscle preservation (dorsal aspect); **B**. MGUH 30414 impression of doublure margin and prehypostomal sclerite (ventral aspect); **C**. MGUH 30405 doublure with terrace lines on left anterolateral margin of cephalon (ventral aspect, orientation WNW); **D**. MGUH 30398 marginal rim and fingerprint-like cuticular sculpture on left anterolateral margin of cephalon (dorsal aspect, orientation ENE); **E**. MGUH 30388 disarticulated cephalon with strong compactional folds. For abbreviations see Table 1 (dorsal aspect).

**Figure 3:**
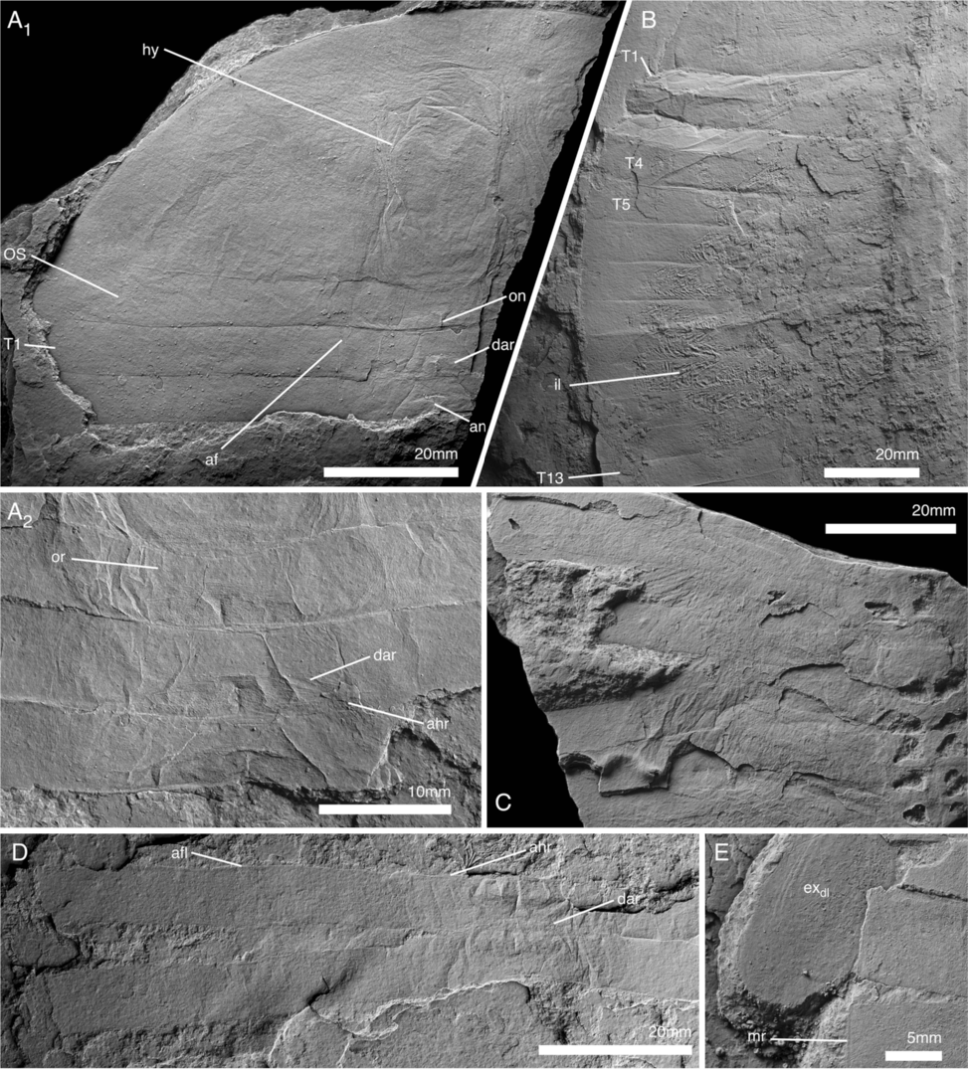
**Tergite articulations of *Arthroaspis bergstroemi *n. gen. n. sp. A**. MGUH 30400, specimen with cephalon and two articulated thoracic segments; **A**_**1**_ (dorsal aspect); **A**_**2**_ (ventral aspect); **B**. MGUH 30407 (pt), specimen with tergites tilted relative to each other, indicating tergite articulations interconnecting tergites along their entire width. Inner lamella is preserved (dorsal aspect); **C**. MGUH 30390 articulated thoracic tergites and appendages, tergites partially disarticulated (dorsal aspect); **D**. MGUH 30402 two adjacent tergites articulated at the half-rings only (ventral aspect); **E**. MGUH 30389, lateral gape of adjacent tergites, with narrow marginal rim on tergites and displaced distal lobe of exopod (dorsal aspect).

**Figure 4:**
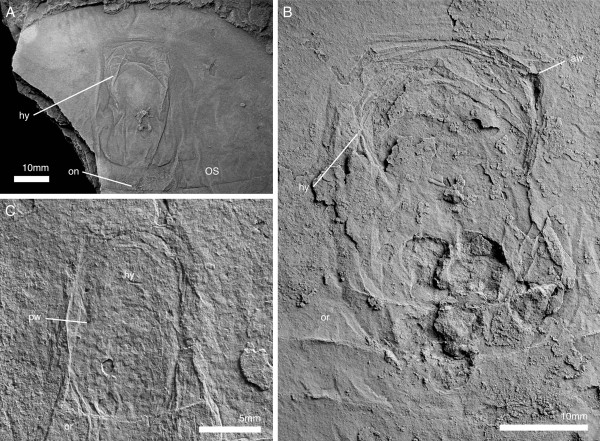
**Hypostome of *Arthroaspis bergstroemi *n. gen. n. sp. A**. MGUH 30413, impression of hypostome under cephalon, with box-like wrinkling at the anterior margin (dorsal aspect); **B**. MGUH 30416 outline of hypostome with impression of anterior wing and box-like wrinkling of the cephalic shield above (ventral aspect); **C**. MGUH 30387, outline of hypostome with impression of posterior wing (ventral aspect).

**Figure 5:**
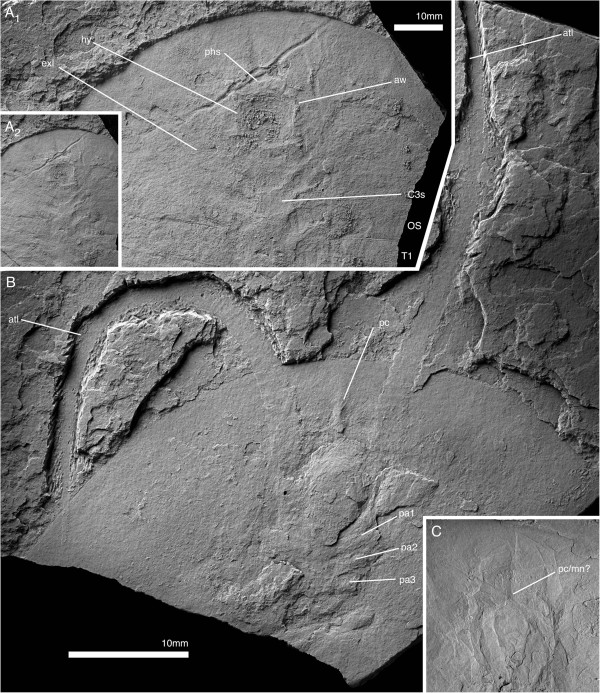
**Ventral cephalic structures of *Arthroaspis bergstroemi *n. gen. n. sp. A**. MGUH 30417, showing impression of hypostome and cephalic sternites (dorsal aspect); **B**. MGUH 30391, showing the insertion of cephalic appendages (ventral aspect); **C**. MGUH 30407 (cpt), close up of anterior glabellar region, showing preglabellar crease and possible median node.

*Arthroaspis bergstroemi* is one of the largest organisms in the Sirius Passet Lagerstätte; the largest specimens are over 210 mm in length. In outline it is subrectangular, with distinctly delimited cephalic, thoracic and pygidial regions. The cephalon of *A*. *bergstroemi* is a large, semi-elliptical shield that lacks genal spines and bears a glabella (Figures 1A, 2A, 3A). The hypostome is rounded and rectangular to oval in outline, longer than wide (Figures 2E, 3A, 4, 5). Its total length is about one third of total cephalic length; the total width is one fifth of the total cephalic width. Its maximum width is in the anterior quarter at the forward-projecting anterior wings (Figure 5A). Its lateral margins curve outward posteriorly to form posterior wings (Figures 2E; 4C); the antennular insertion (see below) is at a notch between the anterior and posterior wings, recessed under the median body (Figure 5B). Anteriorly, the hypostome is bounded at a transverse suture by an arcuate prehypostomal sclerite with an anteriorly diverging, raised central region (Figures 2B; 5A). The prehypostomal sclerite is well-separated from the cephalic margin and doublure (Figures 2A, B; 3A); together, the hypostome/prehypostomal sclerite complex is natant (Figures 2E; 3A; 4; 5A). Some specimens show a preglabellar sagittal crease directly in front of the hypostome in the dorsal cephalic shield (Figures 1B; 2A, E). The final segment of the cephalon is somewhat differentiated (Figures 2A, B, E; 3A), especially in the axial region where the glabella is marked by a furrow bearing a poorly-developed occipital node (Figures 2E; 3A). The occipital furrow continues laterally into the cephalon away from the glabella, but dies out at the margin. The margin of the cephalon is delimited by a very narrow but distinct border, less than 2% of the length of the cephalon (Figure 2B–D). There is a narrow doublure that bears terrace lines (Figure 2C).

**Figure 6:**
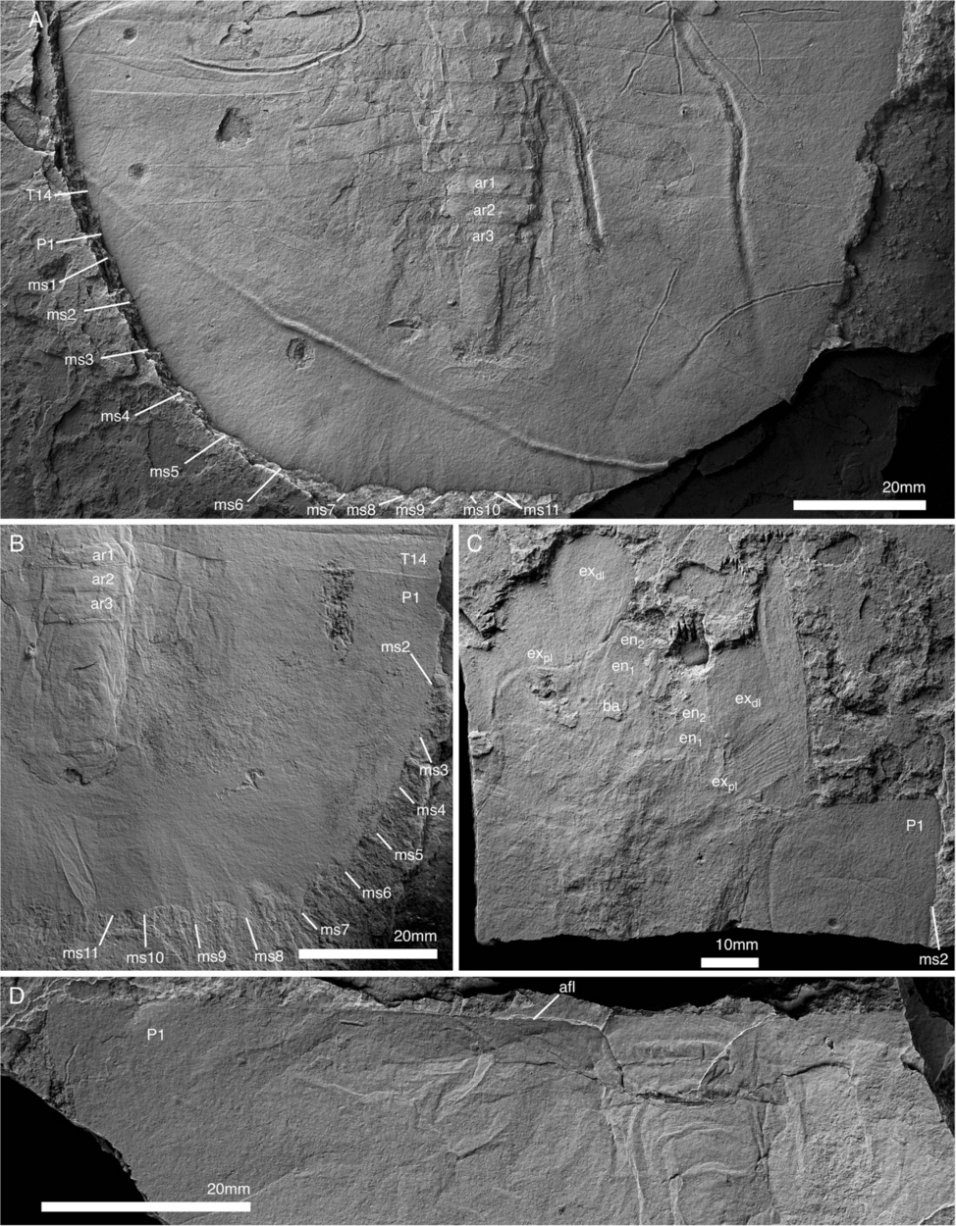
**Pygidium and posterior thorax of *Arthroaspis bergstroemi *n. gen. n. sp. A**. MGUH 30401, pygidium with posterior trunk tergites showing axial rings and segmental marginal spines of pygidium (dorsal aspect); **B**. MGUH 30418, pygidium with posterior trunk tergites showing axial rings, segmental marginal spines, and anterior segment of pygidium (dorsal aspect); **C**. MGUH 30386, disarticulated pygidium with appendages (dorsal aspect); **D**. MGUH 30393, anterior margin of disarticulated pygidium showing anterior segment of pygidium with articulating flanges (dorsal aspect, light from SE).

The thorax consists of 14 trilobate tergites with clearly defined transverse tergal boundaries (Figures 1; 2A). The narrow axis occupies about one fifth of the total width (Figures 1, 2A, 3A, D), laterally demarcated by a weak axial furrow. The axial rings carry broad based axial nodes in their posterior third (Figure 3A) and a crescentic doublure with reticulate cuticular sculpture ventrally, which covers the posterior third of the total length (Figure 3A). The main articulation device is a short (sag, less than one fourth of the tergal length) crescentic articulating half ring (Figure 3A, D). The half ring is not set off by an articulating furrow, but is, rather, laterally confluent with a very short (sag.) flange along the anterior margin of the tergopleura (Figure 3D). The tergopleurae articulate edge to edge (Figures 1A, B, 2A). The outer tergopleura is more distinctively sloping than the inner (Figure 3D), but is defined by a gradual change in slope at about one third the tergopleural width from the axis, rather than possessing a well-developed fulcral line. The tergites bend around laterally at the shelf-like margin to form a narrow doublure (<2% Figure 3D). The ventral side is covered by an inner lamella (Figure 3B). The lateral extremities of tergites bear minute pleural tips that increase in relative length in posterior segments (Figure 6A).

The pygidium takes up slightly more than one third of the total length of the trunk (Figure 1A). The Anteriormost incorporated segment (trunk segment 15) shows traces of a segment boundary, mostly adaxially: the expression of the boundary is weaker, however, than the fully-expressed boundaries of the free trunk segments (Figure 6A). The margin of the pygidium bears well-developed spines, corresponding in morphology and spacing to the tergopleural tips of the trunk tergites and are interpreted as segmental. The spines indicate the presence of altogether 11 segments in the pygidium (Figures 1A; 6A, B). Three axial rings are developed in the pygidial axis (Figure 6A, B). The postaxial portion of the pygidium is a plate-like inversely teardrop shaped strip, laterally delimited by the 11th marginal spine pair (Figure 1A).

**Figure 7:**
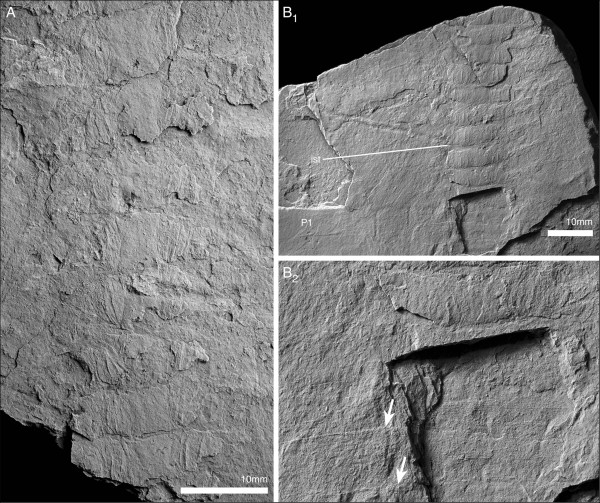
**Sternites of *Arthroaspis bergstroemi *n. gen. n. sp. A**. MGUH 30410 (pt), ventral side of thorax, showing hourglass shaped sternites with sclerotized half-rings in adjacent arthrodial membrane of body-limb joints (ventral aspect, light from NE); **B**. MGUH 30392 ventral side of thorax and anterior pygidial region, showing sternites, body-limb joints and intersegmental tendon (ventral aspect). **B**_**2**_ detail of internal sculpture of cuticle of pygidium, cf Figure 3A; note segmental boundaries in inner lamella (arrows).

All postantennular segments, including the first cephalic one, carry sternites ventrally (Figure 6A). The sternites are hourglass shaped with a narrow convex rim laterally, bounding a weak central depression with an axial ridge. The anterior and posterior margins are straight, and the posterior margin slightly narrower than the anterior (Figure 7A). The sternites decrease moderately in width, more strongly in length towards the posterior of the animal (Figure 7A). Sternites in the trunk directly underly the tergal boundaries (Figures 5A, 7B).

**Figure 8:**
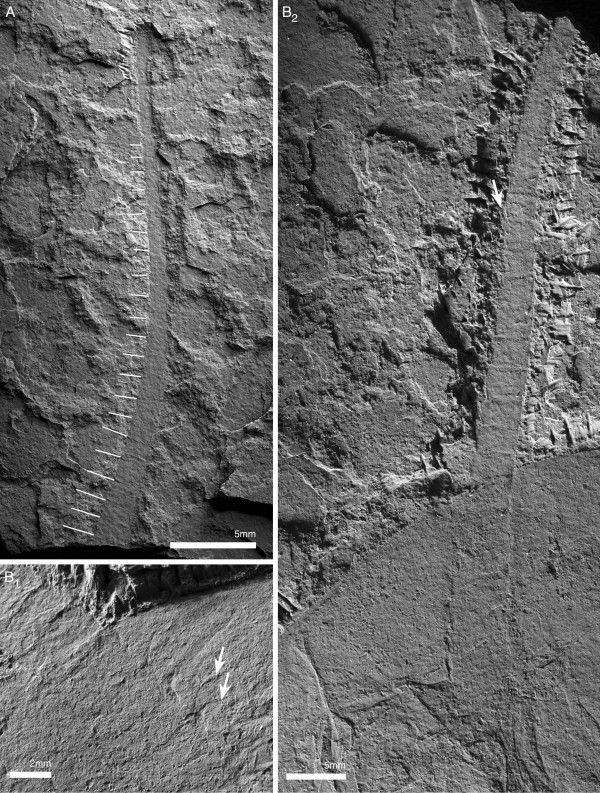
**Antennula of *Arthroaspis bergstroemi *n. gen. n. sp. A**. MGUH 30403 isolated antennula with at least 21 discernible joints (white lines; light from NE); **B**. MGUH 30399, (dorsal aspect). **B**_**1**_, proximal part of left antennula; short articles with biserial spines distally (arrows); **B**_**2**_, right antennula with biserial armature on distal part (arrow).

The antennula is filiform (Figures 5B; 8), consisting of a single branch of well in excess of 20 articles (Figure 8A). The proximal portion is stout, reaching one third of the width of the hypostome (Figure 5B). Articles are short (two times wider than long) proximally, and increase in relative length distal to the cephalic margin (longer than wide; Figures 5B; 8). The articles are tubular, with a slight mediodistal extension carrying biserially arranged spines (Figure 8).

**Figure 9:**
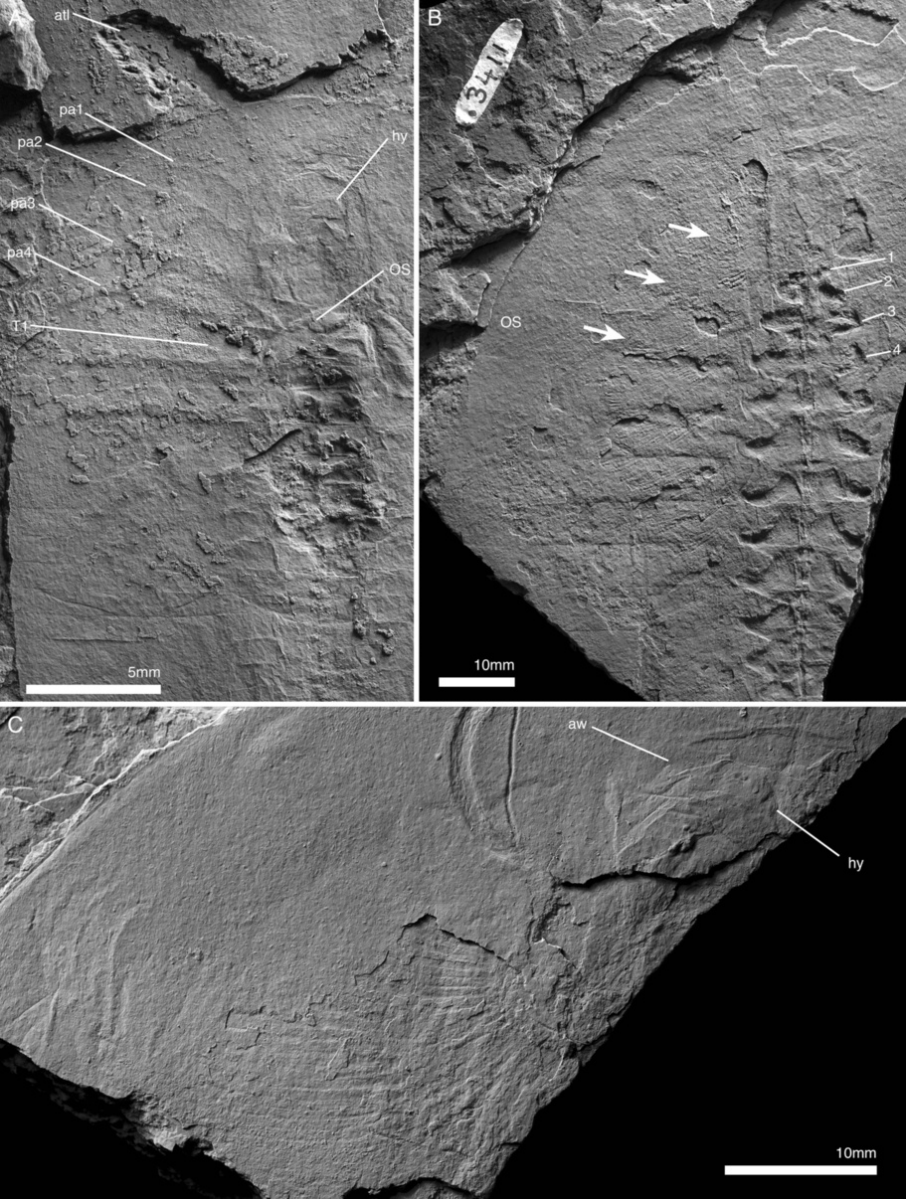
**Cephalic segments and appendages of *Arthroaspis bergstroemi *n. gen. n. sp. A**. MGUH 30412, cephalon and anterior part of thorax; impressions of postantennular limbs, of which three are associated with the cephalon (ventral aspect, light from NE; a cephalon of *Buenellus higginsi* is superimposed on the axis at the cephalothoracic boundary); **B**. MGUH 30397, cephalon and anterior thoracic region showing midgut-structures axially and impressions of the exopods abaxially (arrows; dorsal aspect); **C**. MGUH 30398, fragmentary cephalon, showing impressions of exopod lamellae of the cephalic appendages (dorsal aspect).

**Figure 10:**
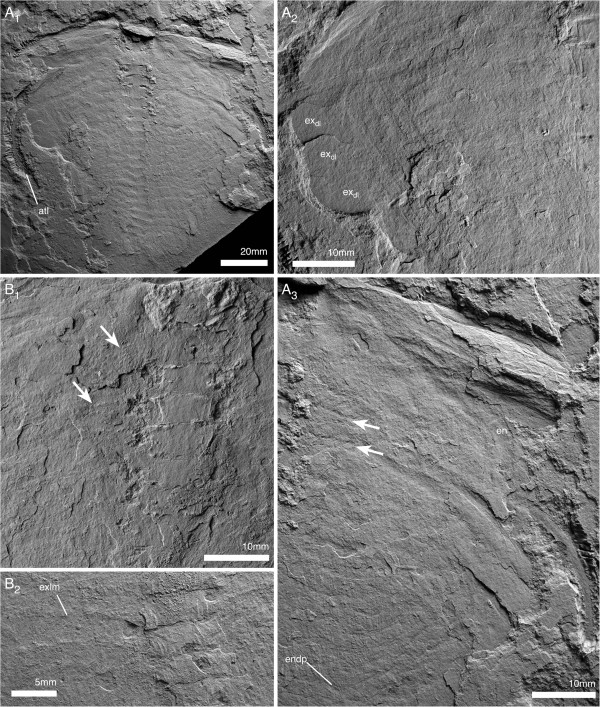
**Ventral side of thorax and pygidium of *Arthroaspis bergstroemi *n. gen. n. sp.** MGUH 30406, specimen with ‘folded over’ cephalon, part and counterpart. **A**. ventral aspect; **A**_**1**_, overview showing sternites and extent of appendages; **A**_**2**_, close up of abaxial region, showing distal lobes of exopods; **A**_**3**_ close up, arrows point to spines on basipod; **B**. counterpart to specimen in **A**; **B**_**1**_ detail of midventral region with sternites and basipod spines (arrows); **B**_**2**_, sternites and impressions of exopod lamellae.

Three appendage-bearing postantennular segments are incorporated into the cephalic region (Figure 9A). The cephalic appendages increase in size posteriorly. The insertions of the anterior cephalic limbs are rotated around the posterior margin of the hypostome (Figure 5B), and the third postantennular limb inserts transversely, as do the trunk appendages (Figure 10A).

**Figure 11:**
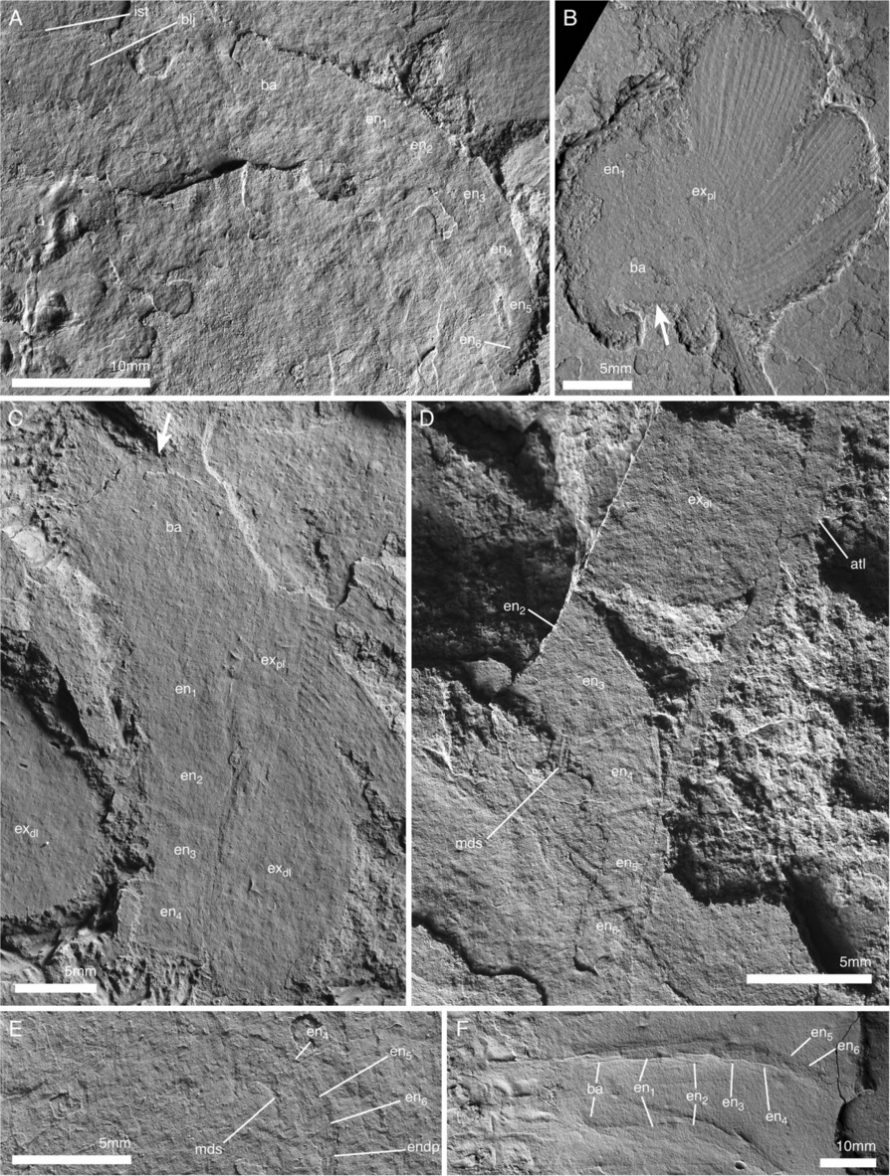
**Postantennular appendages of *Arthroaspis bergstroemi *n. gen. n. sp. A**. MGUH 30383, limb axis with body–limb joint; **B**. MGUH 30394, isolated limb showing proximal part of limb axis with basipod and first endopod podomere, and proximal portion of exopod, arrow marks the proximal margin of the basipod; **C**. MGUH 30419, proximal part of isolated limb, showing large, inward rotated basipod endite and four endopod podomeres; **D**. MGUH 30395, podomeres 3–6 of endopod with mediodistal spines on podomeres three and four; **E**. MGUH 30396, distal portion of endopod, showing distal podomere; **F**. MGUH 30409, impressions of limb axes with boundaries of basipod and seven podomeres discernible (ventral aspect).

The body–limb joint of the trunk appendages consists of extensive arthrodial membrane with three to four weakly sclerotized half-rings (Figure 10B_2_). The basipod carries the proximal portion of the exopod laterally, and the endopod distally to laterodistally. The median edge of the basipod is drawn out into a single endite (Figures 6C; 7B_1_; 11B) armed with biserial spines (Figure 10A). The endopod has seven podomeres (Figures 11A, E–F); en7 forms a distal claw (Figures 10A_3_, 11E). en1 articulates with the proximal portion of the exopod laterally, and is drawn out into a spinose endite medially (Figure 11B). The median edges of en2–4 are drawn out into spinose endites (Figure 11D). The mediodistal extremities of the podomeres carry mediodistal spines (Figure 11D). Laterodistal spines or other armature at the distal margins of podomeres such as denticles are absent (Figure 11D). The length of the endopods roughly corresponds to or slightly exceeds the width of the tergopleurae in the anterior trunk (Figures 1A; 2A; 9A; 11F) and decreases rapidly in the posterior third (Figure 1A).

The lateroproximal extremity of the exopod slightly extends beyond the proximal margin of the basipod (Figure 11B). The exopods are bilobate flaps consisting of two articles (Figure 6C); the proximal is elongate, carries more than 35 lamellae on an arched lateral edge (Figures 3C; 6C; 9C; 11B), and inserts in the lateral edge of the basipod and en1 (Figure 11A–C). The length of the lamellae corresponds to the maximum length of the distal article (Figure 6C). The distal article is teardrop-shaped to oval and fringed with minute marginal setae (Figures 3E; 6C; 10A_2_; 11D; 12); it articulates with the proximal portion of the exopod, articulation with the endopod is unclear. The marginal setae are roughly the length of the width of the lamellae.

**Figure 12:**
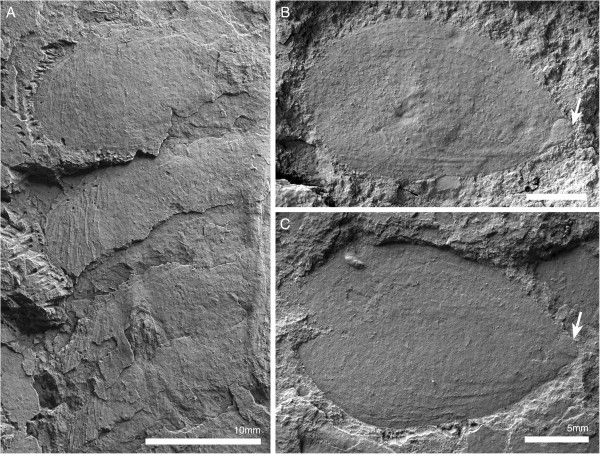
**Distal lobes of exopods of *Arthroaspis bergstroemi *n. gen. n. sp. A**. MGUH 30410 (cpt), left side of thorax of large specimen that preserves imbricating exopods and limb axes three dimensionally on different layers of sediment, note lateral compactional folds (dorsal aspect); **B**. MGUH 30384, isolated distal lobe with setal fringe, lateral side down. Main joint on side opposite arrow, arrow points to possible articulation site with endopod; **C**. MGUH 30385, isolated distal lobe with setal fringe, lateral side down. Main joint on side opposite arrow, arrow points to possible articulation site with endopod.

### Remarks

Apart from impressions of appendages (Figure 9A), evidence for the presence of four post-ocular segments in the head is derived from the cephalic midgut diverticula. There are four pairs of diverticula, the most anterior one being differentiated (Figures 1A, 3C, 9B). Differentiation of the most anterior diverticula pair is known from e.g. Nektaspida [69,91] and *Emeraldella brocki*[31]. In *Arthroaspis bergstroemi* n. sp., the most anterior pair of diverticula is reduced, as in *Misszhouia longicaudata*. The most posterior diverticula pair is in the occipital ring (Figures 1A; 9B), providing evidence that this portion of the glabella and the adjacent strip of tergopleural region set off by a sharp furrow are the dorsal expression of the last segment incorporated into the head, here called the occipital segment. A similar dorsal expression of the last cephalic segment is present in many trilobites that have an occipital ring axially and posterior cephalic border and border furrow abaxially [55]. Alternatively, the posterior border and border furrow might represent the pleural furrow and thereby an intra- rather than intersegment boundary.

The axis of cephalon and trunk tergites must have been differentiated from the tergopleural areas by a change in convexity, as indicated by folds along the axial margin (Figure 2A). These folds often overprint the axial furrow. A similar compactional effect is observed in *Sinoburius lunaris*, where the specimen figured by L-h. Luo, S-x. Hu, S-s. Zhang, Y-h. Tao in their plate 2 figure 4 [92] displays a clear axial furrow, while it is entirely obscured in specimens figured by X-g. Hou and J. Bergström in their figure 77 [12].

At about one third of the tergopleural width from the axis, there is a curvature of the tergopleurae, indicating a change in slope. There is, however, no clearly defined fulcral line as observed in *Siriocaris trollae*[93] or in most trilobites, and the inner tergopleura of *Arthroaspis* does not appear to have been horizontal as in those taxa.

Thoracic tergite articulations are functional in the sense that they contain arthrodial regions separating individual tergites. Tergites are not ankylosed to a single, sclerotized shield. This is evident in the common occurrence of disarticulated material. Frequently disarticulation happens at the cephalon-trunk boundary (Figure 2E) between the thorax and pygidium (Figure 2A) but even thoracic tergites show partial (Figure 3C) or full disarticulation (Figure 3A, D). Often, adjacent tergopleurae are tilted relative to each other, but remain connected, indicating a membranous connection between the individual tergopleurae (Figure 3B) all the way to the lateral extremities. While the tergites thus were clearly separate, it is questionable if the tergite articulations allowed for much, if any, axial flexing of the trunk. Considering the convexity and division in inner and more sloping outer tergopleurae, ventral flexing would only be possible if the outer tergopleurae were imbricating and overlap could have increased during flexing. But since the outer tergopleural margins abut entirely edge to edge, with no signs of overlap in any of the specimens, ventral flexing, for example enrolment, was unlikely. If the inferred presence of a membraneous connection to the lateral extremities is correct, even dorsal flexing, leading to splay of the outer tergopleurae, would have been impossible. Lateral flexing must have been equally impossible. In conclusion, except when moulting, the whole tergum must have functioned more or less as a single shield, as has been inferred for *Skioldia aldna* and *Saperion glumaceum* and the anterior part of the tergum of *Kuamaia lata* and *Helmetia sparsa*[26,88]. In particular for the latter taxa, much weight in the interpretation of non-functional (as in non-rotating) tergite articulations has been laid on the presence of anterolaterally reflexed margins of the anterior tergites. Such anterior flexure does occur in specimens of *Arthroaspis* where cephalon and the larger part of the trunk are articulated (Figures 1A; 2A), but not where only a few tergites are articulated with the cephalon (Figure 3A). We suggest that the anterior flexure is a compactional artefact, owed to distortion of tergite boundaries upon compaction of the semi-elliptoid geometry of the tergum into an elliptical geometry. This has likely also happened in *Kuamaia*, *Skioldia*, and *Saperion* of which disarticulated material is unknown. The absence of disarticulated specimens in these taxa could be because of a genuine absence of functional articulations or merely the paucity of specimens; in *Arthroaspis*, the majority of material is articulated, and given the abundance of the species, the absence of disarticulated specimens in the other taxa could be a statistical effect. Even in the articulated specimens of *Kuamaia*, *Skioldia*, and *Saperion*, the anterolateral flexure varies from moderate to absent (e.g. figs 57, 63, 64A, 66 of Hou and Bergström [12] or figure 5:1, 2 of Edgecombe & Ramsköld [26]). We would therefore discourage the use anterolateral flexure of the anterior tergites’ margins as a helmetiid autapomorphy as frequently suggested [19,22,26,32,81].

Laterally, the tergal margins in *Arthroaspis* can appear effaced (Figures 1A; 2A; 3B) while they appear well-defined adaxially. The presence of disarticulating tergites (Figure 3C, D) demonstrates this effacement to be a preservational artefact. The same kind of effacement is present in *Skioldia*, and based on evidence from *Arthroaspis*, it is suggested that the effacement in *Skioldia* is preservational, too. As in *Arthroaspis*, even in *Skioldia*, the lateral extremities of the tergopleurae form minute but free tips (fig. 16:54 of Hou *et al*. [65]).

The hypostome is often preserved by its outline where the glabella collapsed into the hypostomal cavity below. It appears in negative relief in dorsal aspect specimens (Figure 5A), positive relief on ventral aspect specimens (Figures 2E; 3A; 4; 5B). Impressions of the hypostome occur in both articulated specimens and disarticulated cephalic shields. The hypostomal complex can occur displaced relative to the anterior margin of the glabella. The anterior wings of the hypostome project forward and often cause box-like wrinkling of the cephalic shield above the anterolateral corners of the hypostome (Figure 4). The hypostome and prehypostomal sclerite, where preserved, are always in immediate juxtaposition. They were likely fused or separated by a non-functional suture.

The preglabellar sagittal crease is not observed in all specimens and could be a compactional artefact as suggested elsewhere for other taxa [49,94]. It does seem to follow a morphological structure; it is possible that it is caused by a possible median node on the prehypostomal sclerite (Figure 5B, C) as is present in nekatspids [77]. There are also faint elevations on the prehypostomal sclerite lateral to the preglabellar sagittal crease (Figure 5C), which could be homologous to the paired “frontal organs” of nektaspids [77].

The position of the mouth is unknown; the most posterior position would be at the posterior margin of the hypostome. Since the hypostome is followed by the sternite of the first postantennular appendage immediately (Figure 5A), the position of the mouth is unlikely to have been further posterior than that. The hypostome is relatively short and judging from the antennular insertion (Figure 5B) it seems unlikely that the mouth is recessed far anteriorly under the hypostome.

An exact count of antennular articles is not possible because the antennule is never complete and articles are poorly preserved distally. Some 22 articles have been counted (Figure 8A), but that count does not start at the insertion, so proximal articles could not be counted. Joints become indistinct distally, and cannot be counted either, so it is assumed that the total count of antennular articles is well in excess of the countable number.

The overall preservation does not allow detailed description of the structure of the postantennular cephalic limbs, but there is evidence for lamella-bearing exopods at least in the second and third postantennular appendages (Figures 5A; 9B). The relative proportions of endopods (as seen from impressions) and exopods and the orientation of the exopod lamellae are as in the trunk appendages (Figure 9C). All trunk appendages appear to be inserted transversely (Figure 10A). If the position of the mouth corresponded with the posterior margin of the hypostome, insertion of the first postantennular appendage must have been paraoral but hardly preoral (Figure 5B).

**Figure 13:**
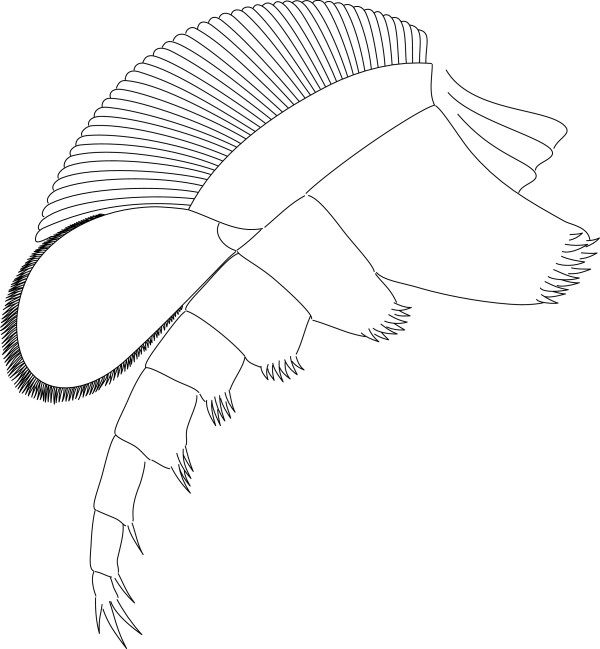
**Generalised reconstruction of trunk limb of *Arthroaspis bergstroemi *n. gen. n. sp.** Generalized reconstruction of a mid-trunk limb of *Arthroaspis bergstroemi*.

A generalised reconstruction of the trunk appendages is given in Figure 13. The appendages appear to change in stance from laterally splayed in the anterior trunk to pendant in the posterior in some specimens (Figure 1A). An isolated appendage (Figure 11C) shows outward curvature of the basipod, as evidenced by the basipod endite that appears rotated relative to the limb axis. Remnants of the margin of the body limb joint indicate the joint to be dorsal to the endopods, but dorsolateral to the basipod-exopod body. It seems that this change in stance is accomplished through change in basipod morphology rather than outward curvature of the proximal endopod podomeres as in *Emeraldella brocki*[31]. In other specimens (Figure 10) even the posterior limbs appear splayed, which might indicate postmortem effects. In either case, judging from what is visible from the proximal portions of the appendages (Figure 10) it seems most likely that further rotation could occur around the extensive membrane of the body–limb joint. Cephalic appendages are consistently splayed to anteriorly extended. The pygidial appendages are not preserved except for their body–limb joints (Figure 10A). The terminal appendages are not preserved, so it is not known whether there were specialized caudal appendages as in e.g. *Olenoides serratus*[87] or *Emeraldella brocki*[31].

The distal article of the exopod is often wrinkled, indicating convexity (Figure 12). However, the massive wrinkling towards the lateral edge in one three-dimensionally preserved specimen (Figure 12A) is likely to be the result from those margins being compressed onto the bedding plane. The distal article of the exopod is sometimes disarticulated (Figure 12B–C), and details of the joint with the proximal article are visible in this disarticulated material. The joint is straight on one side, and embayed on the other. The antero-posterior orientation is not known in the disarticulated material, so it is not clear which side is straight and which embayed. The embayment, which goes across the apex of the proximal corner, could be an indication that the distal article also articulated with the endopod. The distal article seems to be homologous to the two distal articles of the tripartite exopods of *Emeraldella brocki*[31]. The joint with the proximal article in that taxon has the same shape as does its articulation with the endopod, and the median part has setae along its median edge. The character polarity―secondary subdivision of the distal article in *Emeraldella* vs loss of the second joint in *Arthroaspis*―is unclear. A generalised mid-trunk appendage is reconstructed in Figure 13.

**Figure 14:**
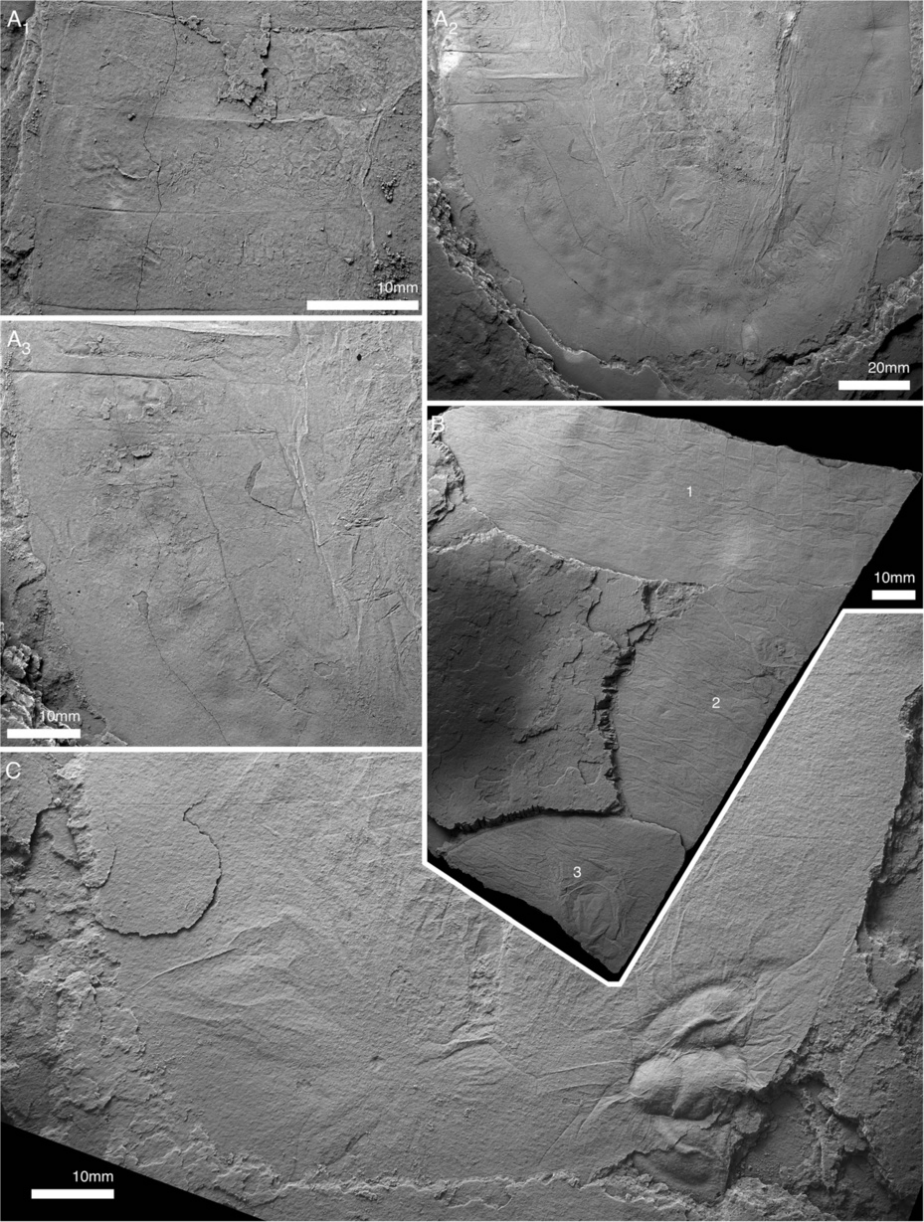
**Inner lamella and tergites in *Arthroaspis bergstroemi *n. gen. n. sp. A**. MGUH 30404, large individual preserving the inner lamella mirroring segmentation; **A**_**1**_, close up of left side of thorax, **A**_**2**_, overview of posterior thorax and pygidium, **A**_**3**_, close up of pygidium. **B**. MGUH 30415, three individuals (numbered) with heavily wrinkled cuticle; **C**. MGUH 30408 posterior of specimen with pygidium draped over isolated cephalon of *Buenellus higginsi*, indicating relatively weakly sclerotized cuticle.

The inner lamella is preserved in only a few specimens (Figures 3B; 14A), and tends to follow the internal segmental organisation of the tergum. This is even the case where the tergites are fused, as in the pygidium (Figure 14A_2–3_).

Tendinous bars are present as transverse intersegmental bars between the sternites, and they extend laterally beyond the lateral margins of the sternites (Figures 7; 10; 11A). Potential muscle preservation as observed in other taxa described from the Sirius Passet Lagerstätte [48,52,95] is observed in some specimens (e.g. Figure 2A).

### Phylogeny

**Figure 15:**
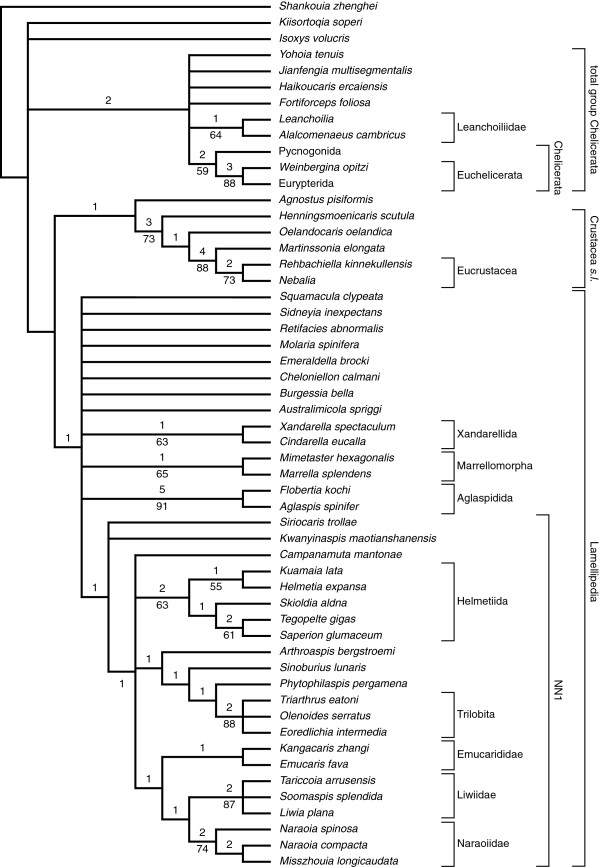
**Unweighted analysis.** Unweighted analysis, strict consensus of 2851 MPTs.

**Figure 16:**
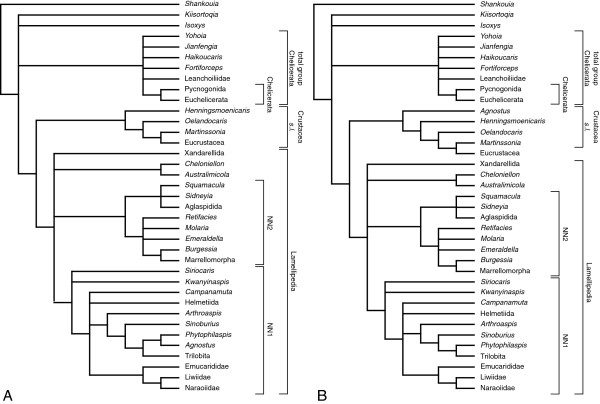
**Weighted analyses. A**. Weighted analyses with *k* = 5–16, strict consensus of 63 MPTs; **B**. k ≥ 17, strict consensus of 63 MPTs.

The unweighted analysis yielded 2851 MPTs (strict consensus in Figure 15). Weighted analyses yielded 21 MPTs for *k*=1, 42 for *k*=2, 20 for *k*=3, and 21 for *k*=4. Analysis with *k*>4 yielded 63 MPTs consistently. Topology remains unchanged for *k*=5–*k*=16 (Figure 16A), and broadly mirrors that of the unweighted analysis, except for increased resolution among basal lamellipedians and a different position for *Agnostus pisiformis* (see below). At *k* ≥17, *Agnostus* is in the same position as in the unweighted analysis (Figure 16B). The topology remains stable with increased *k* values.

Unweighted and weighted analyses retrieve a basal polytomy of *Isoxys*, *Kiisortoqia*, the total group Chelicerata, and a clade of Crustacea *s*.*l*. + Lamellipedia which probably represents the total group Mandibulata. In the unweighted analysis, and at *k*≥17, *Agnostus pisiformis* is the sister taxon to Crustacea *s.l*., whereas it forms a clade with *Phytophilaspis pergamena* which is sister group to Trilobita in weighted analysis with *k*=4–16.

The total group Chelicerata comprises a polytomy of taxa with a short great appendage [95] and the crown group Chelicerata. Autapomorphies for this clade are: 2, first cephalic appendage composed of ≤7 articles (convergent with *Martinssonia* + Eucrustacea); 3, single, large, mediodistal finger on articles of first appendage; 6, elbow joint; 68 (optimized) spines on telson (reversal in crown group Chelicerata). A monophyletic Megacheira ([12]) is not retrieved, but there is no positive evidence for a grade of ‘megacheirans’ leading to the crown group Chelicerata, either [21,23]. We are reluctant to follow [23] and apply the name Megacheira to the whole total group of Chelicerata until relationships are better resolved. Cheliceromorpha [59] also included Aglaspidida, which in the present analysis and that of Ortega Hernández *et al*. [19] is part of the total group Mandibulata. Possible autapomorphies for crown group Chelicerata are: 4, two fingers on short feeding appendage; 7, six postocular segments incorporated into head (prosoma); 8 and 9, exopod on first and second post-antennular appendages absent (convergent in *Cheloniellon*); 22, body limb-joint of arthrodial membrane only (convergent with *Agnostus* + Crustacea *s*.*l*.; problematic because the proximal rings in *Palaeoisopus problematicus* could be homologous to the partially sclerotized half-rings [96]); 31, dorsal median eyes; 35, hypostomal sclerite absent. Further characters that are present in Euchelicerata and have been optimized for Chelicerata: 56, apodous abdomen (possibly with Pycnogonida, see *Palaeoisopus*; convergent with *Yohoia*, *Martinssonia* + Eucrustacea, and *Sidneyia*); 66, keeled styliform telson (present in *Palaeoisopus*).

The clade of (*Agnostus* + Crustacea *s*.*l*.) + Lamellipedia, retrieved in the unweighted analysis and in weighted analysis with *k*≥17, has a number of potential autapomorphies: 3, armature of first cephalic appendage are mediodistal setae; 25, biseral spines or spinose endites along median edge of podomeres 1–4. Characters assumed to be present in the ground pattern but lost in some of the in-group taxa are: 14, exopods differentiated into proximal and distal lobes (*Agnostus* and Lamellipedia, with a reversal in Crustacea *s*.*l*. and modification in Marrellomorpha); 24, endopod of seven podomeres (shortened in Crustacea *s*.*l*.); 66, tubular cap-like to tubular styliform telson (with reversals to plate-like in *Sidneyia* and *Siriocaris*).

The phylogenetic placement of *Agnostus* has been a matter of contentious debate [9,97,98]. As in some earlier analyses [31,60], *Agnostus* is not retrieved as a trilobite but as sister taxon to Crustacea *s*.*l*., but the clade has no jackknifing or Bremer support and is not retrieved in weighted analyses with *k*<17. A problem here is that most characters uniting *Agnostus* with Crustacea *s*.*l*. are only present in the ground pattern of that taxon and lost or modified in Eucrustacea or even earlier in the eucrustacean stem-lineage. Possible synapomorphies of *Agnostus* with the Crustacea *s*.*l*. stem species (represented by the ground pattern) are: 10, cephalic exopods inserting along shorter joint in limbs (character reversal in Eucrustacea is an artefact from character definition, as the exopods insert only in the basipod even in the trunk limbs of the eucrustacean taxa coded here); 11, endopod of first postantennular limb heavily reduced or absent, of second reduced (absent in *Martinssonia* and *Nebalia* because in these taxa the trunk endopods are short); 12, first and second cephalic exopods multiarticulate, each article with mediodistal setae or pair of lateral setae (reversal in *Nebalia*); 19, long spines on exopods (reversal in Eucrustacea); 22, body limb-joint of arthrodial membrane only (convergent with Chelicerata or Euchelicerata). Weighted analysis with *k *≥ 5 <17 retrieve *Agnostus* as sister taxon to *Phytophilaspis*. The clade has no jackknifing support; a potential synapomorphy is character 73, relative length of thorax longer than caudal end. *Agnostus* + *Phyotphilaspis* resolve as sister taxon to Trilobita, with potential synapomorphies: 46, calcitic cuticle; 54, raised axial region of trunk defined by axial furrows; 63, pygidium lacking lateral spines. The clade of (*Agnostus* + *Phytophilaspis*) + Trilobita is lacking jackknifing support.

All ‘artiopodan’ taxa and the Marrellomorpha form a clade that corresponds to the original Lamellipedia [12], though that taxon originally was defined as a grade of stem-chelicerates. Possible autapomorphies would be: 1, first cephalic appendage as antenna (convergent with *Nebalia*, though the latter has a quite different morphology); 2, first cephalic appendage composed of >15 articles (convergently in *Nebalia*; absent in *Molaria*); 18, imbricate exopod lamellae. Further, wide attachment of the hypostomal sclerite (character 35) is the ground pattern state optimized for the lamellipedian node. In the non-lamellipedian taxa included in the analysis, the hypostome is either natant or absent (Chelicerata, following Ortega Hernández *et al*. [19]). Within Lamellipedia, there are multiple reversals to natant, while helmetiids have an autapomorphic state. The analysis does not resolve a monophyletic Artiopoda within Lamellipedia.

Both unweighted and weighted analyses retrieve a clade of *Kwanyinaspis*, *Siriocaris*, and pygidium-bearing 'artiopodans', in the following referred to as NN1. Possible autapomorphies (both convergent with *Agnostus*) are: 50, edge to edge tergite articulations; 55, articulating half-rings and flanges. A possible ground pattern character lost or modified in subsequent nodes is: 16, distal lobe of exopod large, teardrop shaped with long attachment (reversals in Trilobita, *Tegopelte*, and *Misszhouia* + *Naraoia compacta*). A possible autapomorphy for the clade of pygidium-bearing taxa apart from the pygidium (character 59, convergent with *Agnostus* and *Retifacies*) is the absence of a free telson (character 65, convergent with *Agnostus*). The clade consists of a polytomy of Conciliterga, Nektaspida and a clade comprising Trilobita, *Phytophilaspis*, *Sinoburius*, and *Arthroaspis*. It is noteworthy, that among Naraoiidae, well supported within Nektaspida, *Naraoia* is paraphyletic. This mirrors a taxonomic dispute [12,65,69,77] whether or not *Misszhouia* Chen, Edgecombe & Ramsköld 1997 is a valid genus. Despite our result, we refrain from making a statement on the matter given that neither the character set nor the taxon sampling of our analysis is tailored to resolve naraoiid relationships.

*Arthroaspis bergstroemi* itself resolves as the most basal member of a clade including the trilobites. Potential autapomorphies are: 43, distinct trilobation of head shield, defined by axial furrows; 44, Dorsal expression of last segment of head shield (convergent with *Agnostus* in unweighted analysis and with *k *≥ 17). *Sinoburius lunaris* is united with the clade of *Phytophilaspis* + Trilobita by the head shield extending into genal spines flanking the anterior trunk (character 45; convergent with Aglaspidida, a character reversal has to be postulated for *Agnostus* in weighted analysis with *k*=4–16). This placement of *Sinoburius* is at odds with its conventional placement in Xandarellida or Petalopleura Hou & Bergström, 1997 [12,19,22,26,32,81]. Characters proposed as synapomorphies between *Sinoburius* and *Xandarella* + *Cindarella*: eye slits (also *Phyotphilaspis*) [26,81], a head shield overlapping multiple anterior trunk tergites, with small median area of attachment [26] and the presence of an axial spine on a posterior tergite [26]. The last character is problematic, as the spine occurs on the pygidium in *Sinoburius*[92], a subterminal tergite in *Cindarella*[76], and the terminal in *Xandarella*[12]. Homology of this spine is therefore questionable, if not the presence and subsequent loss of a true pygidium were postulated for Xandarellida with the spine being originally on the pygidium and then on different parts of the posterior trunk after the pygidium became split up in Xandarellidae. This seems unlikely when *Luohuilinella rarus,* which seems to lack a pygidium, is taken into account. The presence of eye slits in *Sinoburius*[26] is tenuous, and they are absent in *Cindarella*. Overlap of multiple tergites is present in *Xandarella* and *Cindarella*, but probably not in *Sinoburius*. Edgecombe and Ramsköld [26] cite the holotype as showing the seventh tergite counted from posterior as fully overlain by the head shield, the sixth as partly overlain. The specimen (see also Hou & Bergström figure 77A, B [12]) shows seven free trunk tergites; the head shield is strongly curved adaxially, leaving effectively no overlap with the first (seventh from the posterior) tergite, let alone the second (sixth from posterior). The same is true in the other specimen figured by Hou and Bergström (figure 77C, D in [12]) and that figured by Luo *et al*. (pl. II:4 in [92]) and we assume that the same is the case in the poorly preserved specimens figured by Edgecombe and Ramsköld (figure 1 in [26]). The curvature is an effect of the genal spines bracing the thorax, the synapomorphy with the *Phytophilaspis* + Trilobita clade. The latter clade is defined by charcters 27, the lateral eyes being dorsal (convergent with Euchelicerata and Aglaspidida); 54, raised axial region of the trunk defined by axial furrows (convergent with *Cheloniellon* and *Emucaris*) and the absence of lateral spines on the pygidium (convergent with Tegopeltidae, Emucarididae, and *Naraoia compacta* + *Misszhouia longicaudata*; reversal in *Olenoides*).

Weighted analyses with *k* ≥ 5 resolve a clade herein referred to as NN2. A potential autapomorphy is wide attachment of the hypostomal sclerite (character 35, convergent with *Kwanyinaspis*, *Siriocaris*, and Trilobita; reversal in *Mimetaster*, possibly Marrellomorpha + *Burgessia*). Two addiditional characters are optimized as ground pattern characters of the group, but are known only for few of the included taxa and seem dubious; 17, two joints in flap-like exopod (present in *Emeraldella* and *Sidneya*, while the exopods of *Retifacies* lack joints; unknown for the other taxa). 72, paddles as pre-terminal appendages (known only for *Sidneya* and *Emeraldella*).

## Discussion

### Tergite morphology in NN1

Unweighted and weighted analyses resolve a clade of all pygidium bearing taxa except *Agnostus* and *Retifacies*. *Kwanyinaspis* and *Siriocaris* are united with this clade by tergites that articulate edge to edge with axial articulating half-rings and lateral articulating flanges (characters 50, 55). Previous studies have recognised edge to edge articulations as a potential synapomorphy between Helmetiida and Trilobita [26], but the present results rather strongly suggest this to be a symplesiomorphy. Indeed, tergite morphology is strikingly similar between *Kwanyinaspis*, *Siriocaris*, *Arthroaspis*, and the helmetiids. This has two interesting functional corollaries; first, the articulating half-rings of the mentioned taxa seem to be narrow axial projections of the tergites, while they are well formed devices set off by articulating furrows in the trilobites. The present topology suggests that the trilobite condition is likely derived from the short sagittal projections of taxa like *Arthroaspis*, while articulating flanges and edge to edge articulations are potentially functionally coupled and have already been present in the ground pattern of NN1 as evident by *Siriocaris*[93]. The sophisticated half-rings of trilobites are an adaptation to enrolment [26,99]. The capability to enrol has also been suggested for the Sirius Passet arthropod *Buenaspis forteyi* Budd 1999 [49], where the axis and articulating half-ring take up most of the tergal width and the lateral extremities of the tergopleurae ostensibly have articulating facets [49], as do many enrolling trilobites, but as seem to be absent in the non-trilobite members of NN1. Encapsulated enrolment, i.e. enrolment effectively shielding the ventral side is considered to be a homoplastic feature of derived trilobite taxa [99-101].Various types of encapsulated enrolment have been described [90], and many, though not all of these are functionally linked with a large pygidium. Consequently, the large pygidium, or iso- to macropygous condition, is often considered to have evolved homoplasticly in different trilobite taxa from the supposed plesiomorphic micropygous state, i.e. a small pygidium, in the ground pattern of Trilobita [99]. This seems counterintuitive at first glance, when taking the pygidium bearing clade within NN1 into account; all taxa including the trilobite’s immediate sister taxon and stem, are iso- to macropygous. But with the notable exception of Eodiscina, Kobayashi, 1939, the stratigraphically old trilobite taxa are indeed almost universally micropygous. Basal trilobite phylogeny remains a matter of debate [102], but the basal split, as best supported would be among micropygous Olenellina [103] and all other trilobites, including a paraphyletic “Redlichiina” at the base [104]. The “redlichiine” trilobites equally are micropygous, while the isopygous Eodiscina are considered to branch off above, although some have suggested them to be derived from olenellines [105]. Either placement would support the hyposthesis of the micropygous condition as the ground pattern state for Trilobita. Thus, the relevant questions are; what drove the evolution of large pygidia basally in NN1?, and why the reversal in the stem species of trilobita? Reduction of number of articulations in order to reduce the risk of intersclerite rupture has been suggested as a reaction to selective pressure in eutrilobites driving caudalization [99,106], and this would be equally true for animals with limited ability to flex or enrol. Indeed, the disarticulated material of *Arthroaspis* indicates that the articulation style of basal members of NN1 was more prone to intersclerite rupture, though it is not clear if the preserved cases are postmortem or exuvial. The early trilobites, in contrast to taxa with the plesiomorphic articulation type, could enrol, albeit not encapsulated, and it is possible that the initial loss of the large pygidium in Trilobita is linked to the ability of incipient enrolment, potentially coupled with biomineralization in order to strengthen the tergites for protection. But this remains speculation until more is known about the stem lineage of Trilobita.

One aspect of trilobite tergite morphology that has recently garnered attention is the mismatch of tergite and segment boundaries [107]. There is no direct evidence of the presence or absence of that mismatch in many of the taxa coded in the analysis. There is evidence for *Leanchoilia illecebrosa* where a specimen documented by Y. Liu, X-g. Hou, and J. Bergström in their figure 2D [80] shows very clearly how the appendages insert under the tergite boundaries. A specimen of *Alalcomenaeus cambricus* that shows the relations between tergites and limb insertion best is figured by D.E.G. Briggs and D. Collins in their plate 2, figure 3 [79] and also shows limbs inserting under tergal boundaries. Reinterpretation of the putative tendinous bars in *Kiisortoqia soperi* as ventral cuticle [107] is somewhat tenuous. One criterium for that was that the putative bars did not extend past the axis, but the specimen in figure 12C by Stein [95] shows them to do just that. *Kiisortoqia* thus does not serve as a model for a fossil arthropod displaying dorsoventral alignment of tergites and segments. According to Ortega Hernández and Brena, *Agnostus pisiformis* does not show the dorsoventral mismatch [107], yet the specimen shown in plate 29, figure 4 of K. J. Müller and D. Walossek [108] clearly shows appendages to insert exactly under the edge-to-edge articulations of the tergites. It is therefore possible that the dorsoventral mismatch is more widely distributed than assumed by Ortega Hernández and Brena and may not necessarily mirror a derived mode of tergite formation in trilobites, helmetiids, and nektaspids.

### Appendage morphology and posture

The appendages of trilobites and many of their lower Palaeozoic allies have long been recognized as being similar in morphology [20,109]. In particular, among Trilobita, Nektaspida, Helmetiida, and Xandarellida, appendage morphology seems to be constant, and their exopod morphology in particular has been suggested a synapomorphy [28,31]. Our topology suggests, that this bilobate exopod morphology with lamellar setae is potentially a ground pattern character for Lamellipedia and experiences modification in some of the lamellipedian taxa, such as e.g. Marrellomorpha, where lamellae insert on individual articles rather than on the proximal lobe of a bilobate flap [61]. The presence of this trilobite-like limb morphology basally in a large clade of non-biomineralizing arthropods suggests that the trace fossils often attributed to the trilobites from the earliest Cambrian, notably *Rusophycus*[110], may be indicative of the behaviour of other members of the clade rather than trilobites themselves, which indeed appear in the geological record somewhat after the trace fossils. However, one aspect that would affect trace fossils is limb stance [111]. Conventionally, the limbs of most lamellipedian taxa have been considered laterally splayed [112], though whether this is a true morphological feature, postmortem effect, or subject to flexibility of the body–limb joint has been debated [113,114]. Laterally deflected limbs could be shown to be present in *Emeraldella brocki*, though the outward curvature is achieved in the proximal endopod podomeres [31], as is also the case in *Sidneyia inexpectans* (Stein in preparation). For the Nektaspida, available evidence suggests that the appendages could swing out at the body-limb joint, but there was no outward flexure ‘hard wired’ into the limb axis morphology [69,77,113]. For *Olenoides serratus*, it seems that the limbs indeed were deflected through outward rotation at the basipod, resulting in the endopods inserting distolaterally rather than distally, as reinvestigation of specimens USNM 65515 and 188573 shows (see Whittington’s plates 11 and 16 respectively [87]). How exactly this is morphologically achieved remains unclear. It is worth noting, that the outward splay is restricted to the anterior trunk region, and that limb stance changes gradually to pending towards the posterior, as is observed in *Emeraldella brocki*, defying the conventional wisdom of the fully homonomous limb series in trilobites and their allies. The pending posterior limbs of taxa such as *Emeraldella* and *Olenoides*, like the rotated anterior ones, insert in an extensive arthrodial membrane, and thus are similar to those of the naraoiids. As an interesting developmental note, these are the appendages of the segments that have been formed the latest during development, and thus are the least differentiated and display an ontogenetically early morphology. A similar outward rotation at the basipod as in *Olenoides* seems to be present in *Arthroaspis*, as indicated by the relatively inward turned basipod endite in the isolated limb in Figure 11C. This suggests, that the trilobite-type limb stance was present in the ground pattern of the clade *Arthroaspis* + (*Sinoburius* + (*Phytophilaspis* + Trilobita)).

## Conclusions

*Arthroaspis bergstroemi* is a new arthropod from the early Cambrian Sirius Passet Lagerstätte of North Greenland that displays an interesting combination of characters, with a broadly helmetiid-like tergum, a nektaspid-like configuration of the hypostome and prehypostomal sclerite complex, and trilobite-like features of the head shield and stance of the appendages. Cladistic analysis resolves a monophyletic Lamellipedia as sister taxon to Crustacea *s*.*l*. (possibly representing Tetraconata), lending further support to the notion that the trilobite-like arthropods belong with Mandibulata [19,28,29,31] rather than with Chelicerata as postulated under the traditional Arachnomorpha concept [20,22,27]. *Arthroaspis* falls basally within the clade including the trilobites, *Phytophyilaspis*, and *Sinoburius*, indicating that its helmetiid-like tergum morphology represents the plesiomorphic tergite morphology of a large clade including the pygidium bearing Helmetiida, Nektaspida, and Trilobita. The sophisticated articulating devices of the trilobites are derived from the helmetiid-like tergite articulations, already established in the sister taxa to the pygidium bearing clade. Causes for their evolution remain a matter of speculation, but a functional coupling to the evolution of the ability to enrol is one possible factor. The trilobites' stereotypical lateral splay of appendages, where the endopods insert almost laterally rather than distally on the basipods of the anterior trunk limbs was present in the trilobites closest relatives, including *Arthroaspis*, but not at deeper nodes in the Lamellipdia. This has consequences for the attribution of trace fossils to trilobite-like arthropods.

## Competing interests

The authors declare that they have no competing interests.

## Authors’ contributions

MS prepared, studied, and photographed the material, wrote the initial description, carried out the phylogenetic analysis and wrote the discussion. GEB participated in preparation and study of some of the material and supplemented the description and discussion. JSP and DATH collected the material through several field seasons, organised the material and wrote the sections on the geological setting. All authors have revised and edited the manuscript. All authors have read and approved the final manuscript.

## Supplementary Material

Additional file 1 Mesquite file containing the matrix of 74 characters scored for 54 taxa and trees from the analysis run in TNT under equal weights with 10000 replicates using the ratchet and drift algorithms. Click here for file
